# Comparative transcriptomic analyses of citrus cold-resistant vs. sensitive rootstocks might suggest a relevant role of ABA signaling in triggering cold scion adaption

**DOI:** 10.1186/s12870-022-03578-w

**Published:** 2022-04-22

**Authors:** Amparo Primo-Capella, María Ángeles Forner-Giner, Mary-Rus Martínez-Cuenca, Javier Terol

**Affiliations:** 1 Centro de Citricultura y Producción Vegetal, Instituto Valenciano de Investigaciones Agrarias (IVIA), Valencia, Spain; 2 Centro de Genómica, Instituto Valenciano de Investigaciones Agrarias (IVIA), Valencia, Spain

**Keywords:** Cold stress, RNA-seq, *Citrus macrophylla*, Carrizo citrange, Rootstock, ABA signaling

## Abstract

**Background:**

The citrus genus comprises a number of sensitive tropical and subtropical species to cold stress, which limits global citrus distribution to certain latitudes and causes major economic loss. We used RNA-Seq technology to analyze changes in the transcriptome of Valencia delta seedless orange in response to long-term cold stress grafted on two frequently used citrus rootstocks: Carrizo citrange (CAR), considered one of the most cold-tolerant accessions; *C. macrophylla* (MAC), a very sensitive one. Our objectives were to identify the genetic mechanism that produce the tolerant or sensitive phenotypes in citrus, as well as to gain insights of the rootstock-scion interactions that induce the cold tolerance or sensitivity in the scion.

**Results:**

Plants were kept at 1 ºC for 30 days. Samples were taken at 0, 15 and 30 days. The metabolomic analysis showed a significant increase in the concentration of free sugars and proline, which was higher for the CAR plants. Hormone quantification in roots showed a substantially increased ABA concentration during cold exposure in the CAR roots, which was not observed in MAC. Different approaches were followed to analyze gene expression. During the stress treatment, the 0-15-day comparison yielded the most DEGs. The functional characterization of DEGs showed enrichment in GO terms and KEGG pathways related to abiotic stress responses previously described in plant cold adaption. The DEGs analysis revealed that several key genes promoting cold adaption were up-regulated in the CAR plants, and those repressing it had higher expression levels in the MAC samples.

**Conclusions:**

The metabolomic and transcriptomic study herein performed indicates that the mechanisms activated in plants shortly after cold exposure remain active in the long term. Both the hormone quantification and differential expression analysis suggest that ABA signaling might play a relevant role in promoting the cold hardiness or sensitiveness of Valencia sweet orange grafted onto Carrizo citrange or Macrophylla rootstocks, respectively. Our work provides new insights into the mechanisms by which rootstocks modulate resistance to abiotic stress in the production variety grafted onto them.

**Supplementary Information:**

The online version contains supplementary material available at 10.1186/s12870-022-03578-w.

## Background

As sessile organisms, plants have to adapt to adverse conditions by adopting systemic developmental and physiological alterations that influence the plant genome, proteome and metabolome. Drought, salt and temperature stresses are major environmental factors capable of conditioning plants’ geographical distribution in nature, which limits plant productivity in agriculture and threatens food security [1].

The adverse effects of these abiotic stresses are exacerbated by climate change and global warming, which have been predicted to result in extreme weather episodes with intense rainfall, drought and rising temperatures, along with more frequent cold and hot waves [2–5]. In order to improve plant adaptation to these changing environmental conditions, unraveling the mechanisms that allow plants to sense stress signals and to control plant response to abiotic stresses is a crucial step [6]. Improving plant stress resistance is critical for not only agricultural productivity, but also for environmental sustainability because crops with poor stress resistance consume too much water and many fertilizers [7]. The development of NGS technologies, particularly the analysis of the transcriptome with RNA-Seq, have allowed new insights into the genetic mechanisms by which plant species adapt to their environment under diverse conditions, including exposure to abiotic stresses [8, 9].

Low temperatures have a strong impact on the growth and geographical distribution of plants, and specially affect tropical crops. Plant adaptation to cold stress involves a large number of genes with minor additive effects, and a clear view of the different genetic regulatory pathways regulating responses to low temperatures has only recently been obtained in model plants [10, 11]. During cold acclimation, the expression of cold-responsive (*COR*) genes is activated in Arabidopsis by C-repeat-binding factors, namely genes *CBF1*, *CBF2* and *CBF3*. Recent studies have elucidated the molecular mechanisms by which plants activate COR genes in response to cold signals and have shown that they are also regulated through CBF-independent pathways [12].

*CBFs* are activated by MYC-like bHLH INDUCER OF CBF EXPRESSION 1 (ICE1) [13–15]. Protein ICE1’s stability and, therefore its activity, in the nucleus, increase with the post-transcriptional sumoylation by SUMO E3 ligase SIZ1. During cold stress, stabilization by SIZ1 is counteracted by both ubiquitination and subsequent ICE1 degradation due to ring E3 ligase HOS1 [16], which reaches the nucleus where ICE1 is located [17, 18]. Recent reports show that *ICE1* is regulated by protein kinases: MPK (mitogen-activated protein kinase), MKK (mitogen-activated protein kinase kinase) and MEKK (mitogen-activated protein kinase kinase kinase) [19, 20]. These protein kinases perform different functions and promote the degradation or activation of ICE1 and, consequently, the activation or repression of *CBF* genes. Besides ICE1, there are other transcription factors (TFs) independent of CBF-regulon. In fact, CBF-regulon only represents 12% of the transcriptome response in *Arabidopsis thaliana* at low temperature. Several of these TFs bind *CBF* promoters: *LHY* and *CAMTA* (involved in the circadian clock); *HY5* (involved in light signaling); *SOC1* (involved in flower development); *MYB15* (negative regulator of *CBF* genes) [21, 22].

Experiments run with single, double or triple *cbf* mutants in *A. thaliana* have demonstrated that not all *COR* genes are affected by *CBF* genes, and a CBF-independent pathway is involved in the regulation of cold response genes. Of the 27 first-wave transcription factors, HSFC1, ZAT12, and CZF1 regulate the expression of *COR* genes, but their expression is not affected in *cbf* triple mutants [23–26].

The CBF-COR pathway functions in plants other than *A. thaliana* and *CBF* genes have been identified in a range of plant species like poplar [27], silver birch [28] and several grasses [29]. The conserved role of *CBF* genes has been confirmed in other species like apple, barley, potato and poplar because their overexpression enhances freezing tolerance, even without cold acclimation [23, 27, 30–33].

Light quality, circadian rhythm and photoperiod are other factors that regulate cold acclimation and affect the expression of *CBF* and *COR* genes [34]. Light is necessary for increasing freezing tolerance, and is required for the expression of several cold-regulated genes (*COR*s) in different plant species: phytochromes activate *COR15a* and *COR14b* gene expression, and also regulate CBF target genes under red/far red light conditions [35]. The circadian clock prepares plants for cold stress, and likely occurs at night: at warm temperature, the transcript levels for *CBF1*, *CBF2*, and *CBF3* oscillate and peak at about 8 h after dawn (zeitgeber time 8; *ZT8*) with a trough at about *ZT20*. Moreover, the cold induction of *CBF1, CBF2*, and *CBF3* is “gated” by the clock [25]; if plants are exposed to low temperature at ZT4, the increase in the CBF1, CBF2 and CBF3 transcript levels is much more marked than if plants are exposed to low temperature at ZT16 [36].

A correlation between plant winter hardiness and the accumulation of compatible solutes has been a well-known fact for decades [37] and a growing number of studies indicate that sugars play an essential role in plant cold tolerance [38–40]. Accumulation of soluble sugars, such as raffinose, which in Arabidopsis is involved in stabilizing PS II of cold-acclimated leaf cells [41], or trehalose, which confers high tolerance levels to different abiotic stresses, including low temperatures in rice plants [42], is associated with rapid starch degradation in chloroplasts [43], which would be induced by abscisic acid (ABA) in response to cold and to other abiotic stresses [44].

Cold adaption is often associated with an increased level of this stress hormone, and a rising ABA level is thought to trigger a number of cellular responses required to develop freezing tolerance. Exogenous ABA application results in increased freezing tolerance in various higher plant species, including potato, wheat, winter rape and Arabidopsis, which suggests that ABA and cold might function in common physiological processes and lead to the development of enhanced freezing tolerance in plants [44].

Citrus is one of the most economically important fruit tree crops in the world, with 148.7 million tons registered during the 2018/2019 season (FAO, 2021), which represents a 20.8% increase in the last 10 years and reflects constant annual growth. Climate is the most important factor for site selection in most citrus-growing regions around the world. Beyond certain limits, losses of tree crops or impaired fruit quality as a result of adverse weather conditions are so important that the production of any citrus fruit can be rendered impossible or unprofitable. Freezing temperatures are some of the most limiting factors for citrus production. Temperatures below 0ºC lead to the formation of intercellular ice crystals, which cause dehydration and damage trees [45]. The threshold temperature that kills young citrus shoots is -12ºC [46], but it is common knowledge that citrus species’ cold hardiness considerably varies.

Rootstock/scion combinations are frequently employed in the citrus industry to improve fruit production and quality, as well as tolerance to biotic and abiotic stresses. Rootstocks are selected for root traits linked with resistance to pests and pathogens from soil, but also with several abiotic stresses, such as salinity, drought, floods and cold hardiness. One example of the importance of employing rootstocks is to control citrus tristeza virus (CTV). This virus devastated Spanish citriculture in the 1970s, but it was controlled thanks to the use of tolerant rootstocks [47].

The influence of rootstocks on fruit quality-related traits has a proven significant effect on mandarin fruit size through cell size regulation [48], and also on tree growth, yield and quality, the leaf mineral composition of lemon [49], and even on the flavonoid content of lemon juice [50].

A cold hardiness study conducted with a tetraploid Carrizo citrange rootstock showed enhanced natural chilling stress tolerance for common clementine [51]. Carrizo citrange (CAR) is a widespread rootstock resulted from crossing sweet orange (*Citrus sinensis* Osb.) and *Poncirus trifoliata* (L. Raf.), a citrus-relative species that can withstand temperatures of -26°C when fully cold-acclimated [52]. Several studies have shown that cold acclimation in Poncirus is mediated by the CBF regulon [53], with ICE1 acting as a central regulator of cold response [54]. The PtrbHLH transcription factor, shown to confer cold tolerance to Poncirus [55], is able to promote enhanced cold tolerance when overexpressed in pummelo (*Citrus grandis*) [56]. On the other hand, *Citrus macrophylla* Wester (MAC), which belongs to the papeda group and is the most widespread rootstock in lemon orchards, is extremely sensitive to low temperatures [57–59].

In this work, we used RNA-Seq technology to study how Valencia sweet orange responds differently to cold stress depending on the employed rootstock. We evidence that regulatory processes produce cold acclimation and suggest a mechanism by which Carrizo, a low-temperature resistant rootstock, triggers cold acclimation responses in the grafted scion.

## Results

### Total soluble sugars, starch and proline accumulate in response to cold treatment

**Fig. 1:**
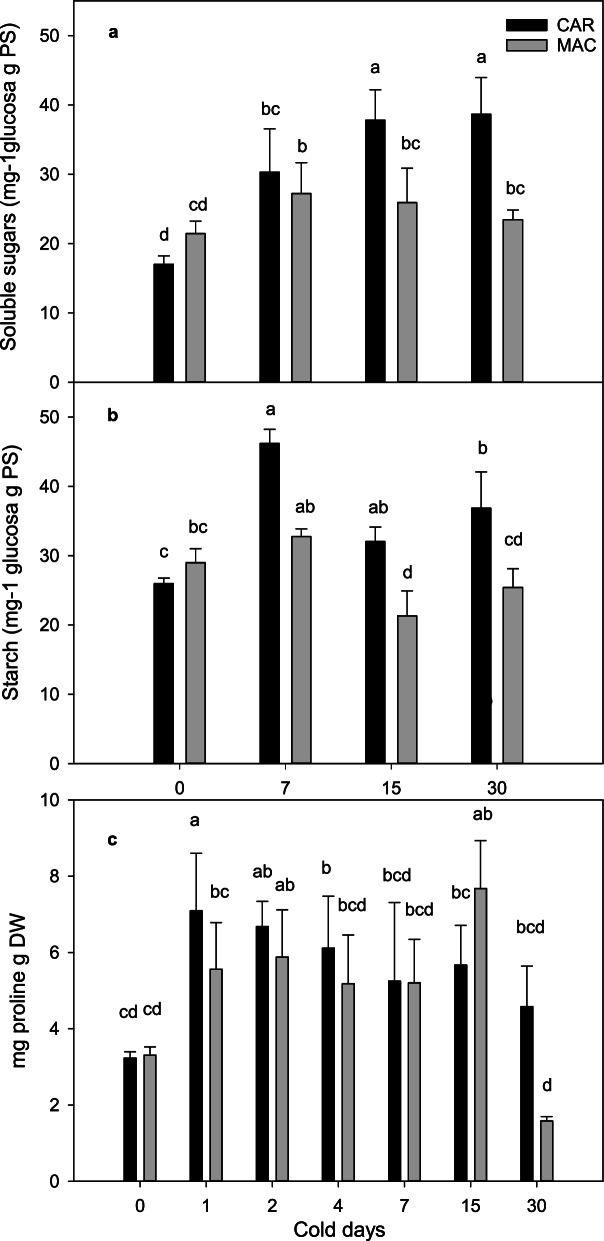
Quantification of osmolytes in CAR and MAC leaves. Plants were grafted and grown under 1°C and control conditions for 0, 15, 30 days. **a** Total soluble sugars, **b)** starch and **c)** proline. Values are the means±SE of three biological replicates (*n*=3) and three technical replicates per biological sample. The treatment effect was tested by a multiway ANOVA. Different letters indicate significant differences (*P* < 0.05) according to LSD

Total soluble sugars and starch were quantified by spectrophotometry methods after 0, 7, 15 and 30 days of cold treatment. A 2-fold increase in total soluble sugars (Fig. 1a) was found in the C30 plants in relation to CAR0. However, the MAC plants only showed a slight increase in total soluble sugars, and no significant difference was found between M0 and M30.

Starch content initially increased in the CAR samples on day 2, and was always higher in the cold-treated plants than in the controls (Fig. 1b). On the contrary, starch content slightly lowered in the MAC cold-treated plants, which had the highest concentrations on day 0 (Fig. 1b).

Free proline was quantified by spectrophometric methods and on days 0, 1, 2, 4, 7, 15 and 30. As expected, proline concentration in leaves increased under cold conditions, and was generally higher in CAR than in MAC, and more significantly so at the end of the experiment with a more than 2-fold increase (Fig. 1c).

A metabolomic profile that comprised some relevant primary metabolites was performed on days 0 and 30 under cold conditions (Table 1). The levels of reduced sugars (rhamnose, trehalose, glucose) were higher on day 30 in both rootstocks, although the increase in CAR was greater than in MAC. The increase in fructose and raffinose was marked, especially raffinose, which was 300-fold in CAR and 400-fold in MAC.

**Table 1:** Relative quantification of the primary metabolites in CAR and MAC leaves at 30 cold days

	Carrizo	*C. macrophylla*	Carrizo	*C. macrophylla*	Carrizo	*C. macrophylla*
	**Glucose**		**Fructose**		**Saccharose**	
Control	1.00±0.140 b	1.11±0.049 ab	1±0.531 b	1.07±0.321 b	1±0.293 b	0.60±0.115 b
Cold	1.14±0.028 ab	1.36±0.161 a	11.53±2.61 a	8.86±1.54 a	2.17±0.289 a	1.92±0.088 a
	**Raffinose**		**Rhamnose**		**Trehalose**	
Control	1±0.145 c	0.62±0.085c	1.00±0.032 bc	0.87±0.029 c	1.00±0.47 bc	0.695±0.048c
Cold	336.60±16.77 a	299.55±10.27 b	1.22±0.075 a	1.13±0.012ab	3.02±0.510 a	2.33±0.561 ab
	**GABA**		**Succinic acid**		**Fumaric acid**	
Control	1.00±0.065 c	1.17±0.175c	1.00±0.119 ab	0.75±0.030 b	1.00±0.025 b	1.14±0.043 b
Cold	3.53±0.661 b	6.09±0.393 a	1.95±0.469 ab	2.08±0.582 a	1.18±0.086 b	1.60±0.178 a
	**Palmitic acid**		**Stearic acid**		**Glycerol**	
Control	1±0.046 b	1.30±0.073a	1.00±0.051 b	1.29±0.069 a	1.00±0.089	1.21±0.059 a
Cold	0.35±0.059 d	0.65±0.039 c	0.37±0.057 d	0.67±0.043 c	0.52±0.056 b	0.68±0.052 b
	**Phenylalanine**		**Proline**			
Control	1.00±0.502 ab	1.52±0.495 a	1.00±0.439 b	0.92±0.094 b		
Cold	0.18±0.074 c	0.56±0.380 ab	3.11±1.740 a	2.22±1.739 ab		

Plants grafted and grown at 1 °C and under the control conditions. The values are the means±SE of three biological replicates (*n*=3) and three technical replicates per biological sample. The treatment effect tested by a multiway ANOVA, and different letters, indicate significant differences (*P* < 0.05) according to LSD

For protein amino acids phenylalanine and proline and non-protein amino acid GABA quantification, no significant differences appeared at 30 days in relation to the control (Table 1). However, some differences were found for total proline when comparing both rootstocks, which was 2-fold higher in CAR than in MAC at 30 days. This confirmed the previous results shown above.

### ABA significantly accumulates in Carrizo roots

**Fig. 2:**
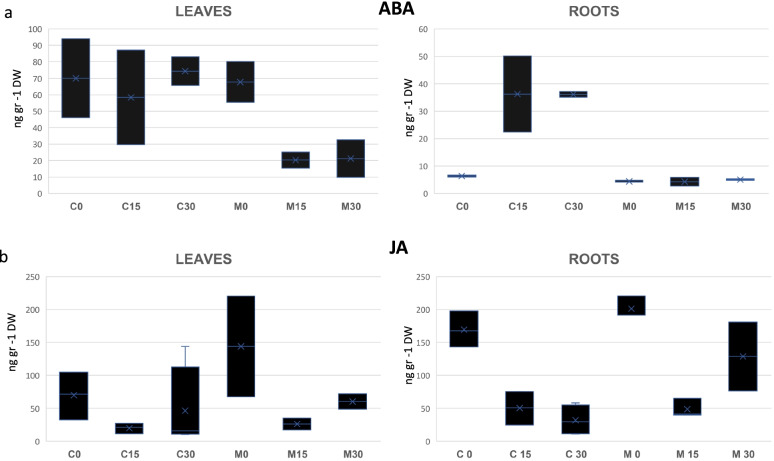
Quantification of ABA and JA hormone in leaves and roots of the CAR and MAC plants grafted in Valencia delta seedless and grown under 1°C and control conditions for 0, 15, 30 days. **a** ABA quantification in leaves and roots, respectively, and **b)** JA quantification in leaves and roots, respectively. The values are the means±SE of three biological replicates (*n*=3) and three technical replicates per biological sample

The quantification of ABA and jasmonic acid (JA) hormones was carried out in roots and leaves at 0, 15 and 30 days (see Methods) and significant differences were found in the accumulation of these hormones in the different organs and rootstocks (Fig. 2).

The ABA concentration in leaves lowered from 90 ng g^-1^ DW in M0 to 20 ng g^-1^ DW measured in M15 and M30, but remained constant in CAR throughout the experiment at a concentration of around 60 ng g^-1^ DW (Fig. 2a). The ABA concentration in roots remained constant at low levels (10 ng g^-1^ DW) in MAC, but increased 3-fold in CAR30. The total ABA concentration in the C30 roots was strikingly higher (40 ng g^-1^ DW) compared to M30 (5 ng g^-1^ DW). The ABA concentration was 8-fold higher in C30 (Fig. 2b).

Initially the JA concentration was significantly higher in the MAC leaves (Fig. 2b), with similar levels in roots in both rootstocks (Fig. 2d). A drop in the amount of JA was observed in the two analyzed organs from C15 and M15. A lower JA concentration continued in C30, while a clear gain in M30 was noted in both roots and leaves.

### *Macrophylla* plants show damage as a result of cold treatment

**Fig. 3:**
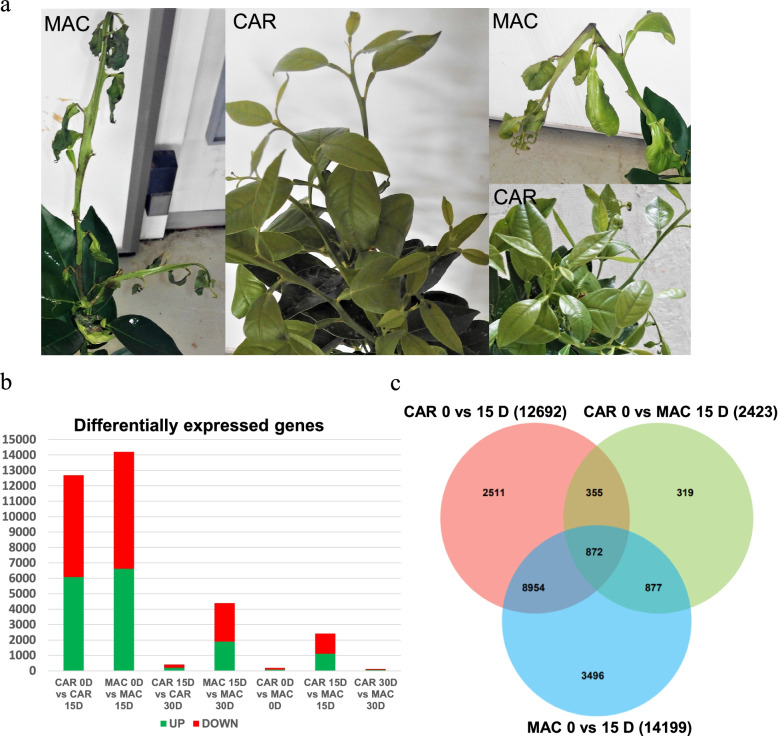
Phenotypic damage in the MAC and CAR plants and the RNA-seq overview. Image **a)** shows the MAC and CAR rootstock-grafted plants at the end of experiment at 30 days of 1ºC. **b** comparison between the intraspecies and interspecies analyses. The number of genes was distributed in the up-regulated transcripts depicted in green and the down-regulated transcripts in red; **c)** Venn diagram representing the common DEGs between the intraspecies and interspecies analyses at 15 cold days

We analyzed the CAR and MAC plants to search for any effects caused by low temperatures after 30 days at 1 ºC. Although the Valencia shoots presented leaf damage in both rootstocks, differences were observed in young shoots, with much severer effects on the scions grafted onto the MAC rootstock caused by cold temperatures. In MAC plants, young shoots showed brown areas and dead leaves, while in the Carrizo ones they were completely normal (Fig. 3a).

### Transcriptomic study and differential gene expression analysis

#### RNA-Seq overview

RNA-Seq was carried out as described in the Methods section. Twelve pair-end libraries were constructed and sequenced with 200 bp reads. After quality trimming, 152.2 million read pairs were obtained with an average of 12.7 million reads pairs per sample, which accounted for 30.6 Gb of useful sequence.

Reads were mapped against *C. clementina* 36454 transcripts, as described in the Methods section. Overall, more than 120 million reads (79%) were mapped against the reference transcripts, of which 77.8 million reads aligned 1 time and 42.2 million reads aligned many times, while 32.2 million reads did not map. The average number of reads per sample was 100 million reads, and the average number of reads mapped per gene was 274.3.

#### The differential expression between Carrizo and *Macrophylla* is higher after 15D of cold treatment

The differentially expressed genes (DEGs) between the Carrizo and *Macrophylla* samples were determined as described in methods

Intraspecies gene expression analyses were performed by comparing samples from the same rootstock collected after 0, 15 and 30 days of cold treatment. Samples C15 and M15 were respectively compared to C0 and M0, and samples C30 and M30 were compared to C15 and M15. The earliest sample was taken as the control. The 15 days comparisons yielded the largest number of DEGs for both rootstocks, with 12691 for CAR and 14199 for MAC, compared to the 409 and 4391 obtained in the 30 to 15 day comparison, respectively. The number of up- and down-regulated transcripts was similar in all the samples (Fig. 3b). The RNA-seq results were validated by RT-PCR, which was carried out with some relevant DEGs selected from the intra- and interspecies comparative analyses (Additional File 1).

In an attempt to identify transcripts involved in the cold tolerance of CAR, interspecies comparisons of the samples from each rootstock collected on the same date were made. Thus, samples C0, C15 and C30 were compared to MAC0, M15 and M30, respectively. The number of DEGs was much smaller than in the previous strategy but, as observed before, the largest number of DEGs (2423) was obtained from the 15-day samples comparison, while the analyses of the 0- and 30-day ones only showed 186 and 119 DEGs, respectively. The number of the up- and down-regulated transcripts was similar in all the samples (Fig. 3b).

As the largest number of DEGs was obtained after 15 days of cold treatment, our analyses focused on these samples, when the response to low temperatures seemed to peak. The identity of the DEGs obtained by both approaches at 15 days was compared and the results are shown in a Venn diagram (Fig. 3c), where a high degree of overlapping is seen.

This way, 9826 common differentially expressed transcripts were found in the intraspecific analyses (M0 and C0 vs. M15 and C15), which accounted for 77% and 70% of all the obtained DEGs, respectively. Similarly, 2104 differentially expressed transcripts from the interspecific comparison of samples M15 vs. C15 were also found in the intraspecific studies, and represents 87% of the identified DEGs. A core group of 872 (366 up-regulated, 506 down-regulated) transcripts was found to be differentially expressed in all three gene expression studies carried out with the 15-day samples.

#### Functional annotation of DEGs reveals the main pathways associated with cold response

The functional annotation of the differentially expressed gene set obtained from the intra- (0 vs. 15 D) and inter- (CAR vs. MAC) comparisons was performed using the BLAST2GO tool [60] and an a functional enrichment analysis was performed with the FatiGO tool. GO terms with the lowest FDR scores that were overrepresented in at least two of the three comparisons were further analyzed (Table 2).

**Table 2:** The 40 most enriched GO terms for the biological process category at 15 days of cold treatment

GO ID	GO Name	FDR		
		CAR-MAC	CAR 0-15 D	MAC 0-15 D
GO:0009409	response to cold	6.14E-12		5.04E-12
GO:0009768	photosynthesis, light harvesting in photosystem I		4.98E-06	1.45E-06
GO:0010200	response to chitin	1.90E-10	7.99E-05	5.24E-06
GO:0006633	fatty acid biosynthetic process		8.94E-04	2.54E-04
GO:0009642	response to light intensity	9.02E-04		3.59E-04
GO:0080167	response to karrikin	1.92E-03	1.26E-06	1.75E-06
GO:0018298	protein-chromophore linkage		2.57E-05	1.39E-03
GO:0055081	anion homeostasis		1.28E-03	9.26E-04
GO:0009744	response to sucrose		2.59E-03	9.90E-04
GO:0009312	oligosaccharide biosynthetic process		2.08E-03	2.43E-03
GO:0042754	negative regulation of circadian rhythm	3.67E-03	2.20E-03	
GO:0009753	response to jasmonic acid		6.28E-03	1.92E-03
GO:0009751	response to salicylic acid	4.55E-10		8.26E-03
GO:0009688	abscisic acid biosynthetic process		8.72E-03	4.88E-03
GO:0006000	fructose metabolic process		1.41E-02	6.69E-03
GO:0071483	cellular response to blue light	1.02E-02	1.46E-02	
GO:0000302	response to reactive oxygen species		2.87E-02	1.19E-04
GO:0000413	protein peptidyl-prolyl isomerization		2.24E-02	6.44E-03
GO:0009816	defense response to bacterium, incompatible interaction		2.75E-02	2.94E-03
GO:0009734	auxin-activated signaling pathway		7.50E-05	3.06E-02
GO:0042631	cellular response to water deprivation	2.65E-05	4.53E-02	7.88E-04
GO:0002832	negative regulation of response to biotic stimulus		2.90E-02	2.35E-03
GO:0019640	glucuronate catabolic process to xylulose 5-phosphate		1.05E-03	3.19E-02
GO:0010109	regulation of photosynthesis	1.43E-02		2.00E-02
GO:0010018	far-red light signaling pathway		3.43E-02	3.02E-03
GO:0031348	negative regulation of defense response		1.44E-02	2.40E-02
GO:0009658	chloroplast organization	3.37E-02		4.72E-03
GO:0009729	detection of Brassinosteroids stimulus	1.91E-02	3.46E-02	8.26E-03
GO:0006116	NADH oxidation		2.76E-04	4.18E-02
GO:0080148	negative regulation of response to water deprivation	1.45E-02		2.79E-02
GO:0006006	glucose metabolic process		1.76E-02	2.55E-02
GO:0006062	sorbitol catabolic process		1.54E-02	3.19E-02
GO:0051164	L-xylitol metabolic process		1.54E-02	3.19E-02
GO:2000038	regulation of stomatal complex development		3.68E-02	1.21E-02
GO:0046890	regulation of lipid biosynthetic process		1.74E-02	3.73E-02
GO:0010207	photosystem II assembly	1.54E-02		4.18E-02
GO:0010114	response to red light	3.18E-02		3.03E-02
GO:0015996	chlorophyll catabolic process	3.21E-02		3.03E-02
GO:0007267	cell-cell signaling		3.46E-02	3.79E-02
GO:0008643	carbohydrate transport	4.57E-02	2.75E-02	

The most significantly enriched biological function was response to cold, with the lowest FDR score. Other overrepresented functional annotations were mobilization of sugars, regulation by ABA and JA hormones, or light and circadian cycles.

We found an abundance of GO terms related to sugar metabolic pathways: response to sucrose, oligosaccharide biosynthetic process, fructose metabolic process, glucose metabolic process, sorbitol catabolic process, etc., Some enriched terms such as the raffinose family oligosaccharide biosynthetic process were present in the interspecific comparison, others like galactose catabolic process were obtained in the C0 vs. C15 analysis (see Table 2).

For light response and the circadian clock, an overrepresentation of terms like photosynthesis, light harvesting in photosystem, protein-chromophore linkage, negative circadian rhythm regulation, far-red light signaling pathway and response to red light, was also observed.

Several enriched biological processes were related to the hormone signaling: response to jasmonic acid, response to salicylic acid, ABA biosynthetic process, the auxin-activated signaling pathway, etc.

Similar results were obtained when searching the KEGG database [61–63] to determine the biological pathways represented among the differentially expressed loci at 15 days (Additional File 2). Clearly the carbohydrate metabolism was the most represented, with 512 loci involved. The reduced sugar metabolism pathways, such as those for sucrose, galactose or fructose, displayed the largest number of differentially expressed loci. Moreover, 263 genes were involved in the environmental adaptation, specifically with plant hormone signal transduction and circadian rhythm, which play crucial roles in the response to cold stress. Similarly, pathways for lipid metabolism with 230, biosynthesis of other secondary metabolites with 216, and amino acid metabolism with 129 genes were well-represented in the set of DEGs at 15 days.

### Analysis of the differentially expressed days at 15 days of cold treatment

A study of 13338 genes, which showed a differential expression after 15 days under cold conditions (M0 vs. M15, C0 vs. M15, M15 vs. C15), was carried out to identify those involved in cold response in citrus and, more specifically, those that could be responsible for the different sensitivity to low temperatures displayed by the two rootstocks. As expected, a significant number of DEGs corresponded to the genes involved in the different pathways associated with cold response, such as the CBF regulon, light and circadian clock regulation, and metabolism of sugars.

### CBF regulatory pathway

**Fig. 4:**
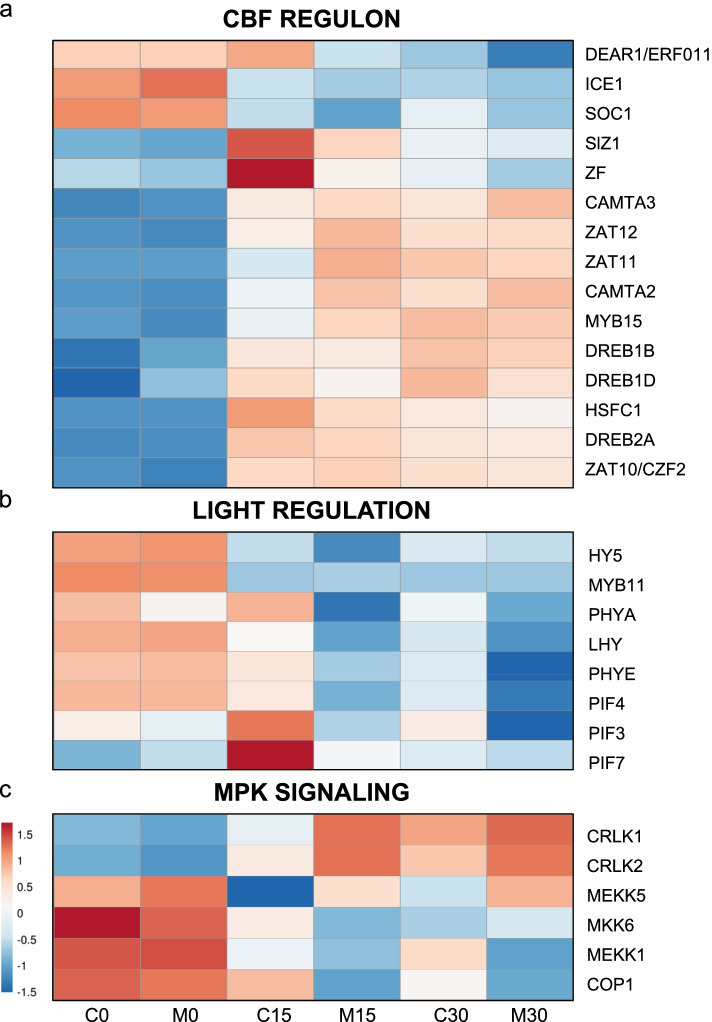
Heat map of the gene expression of the cold-regulated genes in Carrizo and *C. macrophylla* plants at 0, 15 and 30 cold days. **a** from the CBF regulon, **b)** light regulation-related and **c)** MAP kinase signaling

Several genes playing relevant roles in the CBF regulatory pathway were differentially expressed during cold treatment. They displayed different expression patterns, which are shown on the heat map in Fig. 4a.

Three CBF/DRB-like genes were identified, DREB1B (LOC18049462), DREB1D (LOC18033409) and DREB2A (LOC18043521), which were overexpressed in samples C15 and M15 vs. C0 and M0. Interestingly, both fold-change (FC) and the normalized expression levels were generally higher in the Carrizo samples than in the Macrophylla ones, e.g., DREB1D FC in the M0-M15 comparison was 18-fold higher than in C0-C15, and its expression level was almost double.

An ortholog of gene ICE1, LOC18051975, was also identified, which displayed a markedly reduced expression in both samples MAC and CAR. E3 ubiquitin-protein ligase COP1, coded by citrus gene LOC18050526 responsible for ICE1 degradation, showed a steadily decreased expression in both samples CAR and MAC during cold treatment. The SlZ1 citrus gene, LOC18045500, which mediates the SUMO conjugation of ICE1, was differentially expressed in intraspecies comparisons C0-C15 and M0-M15, and its expression on day 15 was significantly higher in the CAR samples.

The closest citrus relative to the negative regulator of cold response, the SOC1 transcription factor located at gene locus LOC18046724, was down-regulated during cold treatment, while the promoter of the CBF genes expression MYB15 (LOC18032541) showed increasing expression levels during the same period.

Downstream of the CBF genes, the citrus relatives of two genes rapidly induced under the cold conditions, ZAT10 (LOC18035015), ZAT11 (LOC18039334), and ZF (LOC18056156), which is involved in COR genes activation, were also differentially expressed with increasing expression levels during the low temperature treatment. A similar expression pattern was displayed by LOC18034062 and LOC18055900, the citrus equivalents of factors ZAT12 and HSFC1, which have also been described as COR genes activators, but independently of CBF activity. In this case however, the expression in the CAR samples was significantly higher than in the MAC ones. Protein DEAR1A, encoded in citrus by locus LOC18036471, belongs to this group, whose expression decreased in M0 vs. M15, which resulted in significantly higher levels of the transcript in C15 vs. M15.

### Light signaling and the circadian clock

The DEGs included some key factors that integrate light and circadian clock regulation with cold response, as shown in Fig. 4b. We identified the citrus ortholog of phytochrome A (LOC18035337), which is an elicitor of the cold response. It was up-regulated only in the C15 samples, and phytochrome B (LOC18033413), which plays an antagonistic role, was down-regulated in all the 15D samples. Other relevant light regulation system members were found to be differentially expressed, such as phytochrome interacting factors PIF3 (LOC18034179), PIF4 (LOC18043439) and PIF7 (LOC18046731) with lowering expression levels.

Citrus LHY gene LOC18047565 was down-regulated during the cold treatment period. However, its expression levels were always much higher in the CAR samples compared to the MAC ones. For example, samples C15 had 4716.40 TMMs, and samples M15 had 1445.42 TMMs.

We also identified a citrus *HY5* ortholog gene, LOC18054790, which showed a slight expression increase in the C15 samples, while remaining unchanged in M15. However, the amount of the *HY5* transcripts was 4-fold higher in the C15 samples compared to M15, and almost 3-fold in C30 vs. M30.

### Calcium and MAP kinase signaling cascades

The heat map in Fig. 4c shows several mitogen-activated protein kinases among DEGs MEKK1 (LOC18040613), MEKK5 (LOC112100736) and MKK6 (LOC18056166), whose expression lowered in M15 and C15 vs. M0 and C0. We also identified two orthologs of calcium/calmodulin-regulated receptor-like kinases CRLK1 and CRLK2, the citrus genes at LOC18054581 and LOC18039195, which had increased expression levels in the 15D samples, but were significantly higher in the CAR ones.

### Sugars and amino acids metabolism

**Fig. 5:**
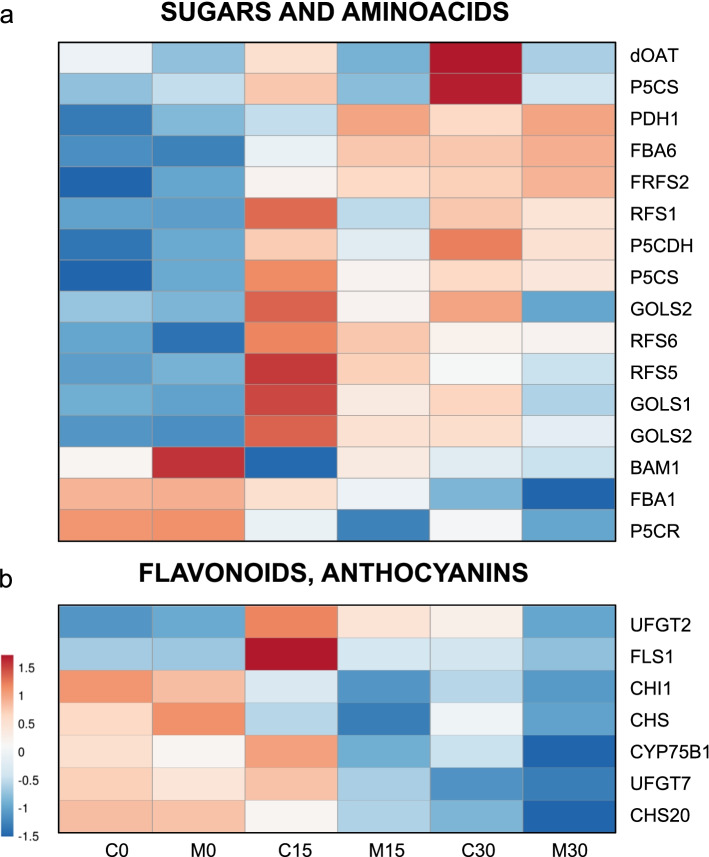
Heat map of the gene expression of the primary and secondary metabolisms in Carrizo and *C. macrophylla* plants at 0, 15 and 30 cold days. **a** from sugars and amino acids-related genes and **b)** the anthocyanins biosynthesis route

According to the changes observed in the accumulation of sugars, dozens of genes related to their metabolism were found to be differentially expressed during cold treatment. The most significant results are shown in the heat map in Fig. 5a.

Raffinose displayed the most remarkable increase in sugar concentration during cold exposure. Several genes coding for key enzymes for raffinose biosynthesis as galactinol synthase, GOLS1 (LOC18044188) and GOLS2 (LOC18040981 and LOC18031271), and raffinose synthase, RFS1 (LOC18031328), RFS2 (LOC18047580), RFS5 (LOC18050892) and RFS6, (LOC18055822) displayed increasing expression patterns with significant FC.

Our analyses also revealed fructose accumulation during cold treatment, and two possible orthologs of enzyme fructose-1,6-bisphosphate aldolase (FBA), which is a key enzyme in sugar metabolisms, but is also involved in the response to abiotic stresses. Two citrus FBA genes were differentially expressed: cytosolic LOC18040008 and chloroplastic LOC18034756. Chloroplastic FBA had similar expression values in samples CAR and MAC, while the cytosolic one displayed a marked expression increase with FCs of 3.15 and 5.02 in the C0-C15 and M0-M15 comparisons, respectively. Gene BAM1 (LOC18048130), coding for β-amylase, which was identified based on its sequence identity with PtrBAM1 from *Poncirus trifoliata*, was also differentially expressed, and showed a reduced expression at 15 days for samples CAR and MAC.

As the most significant change in amino acid quantification was proline accumulation, the expression patterns of the genes involved in proline synthesis were analyzed in detail. They are shown as a heat map in Fig. 5a.

The citrus orthologs of dOAT, ornithine aminotransferase (LOC18051867); P5CS, delta-1-pyrroline-5-carboxylate synthase (LOC18038188, LOC18044634); PDH1, proline dehydrogenase 2 (LOC18039477), P5CR, pyrroline-5-carboxylate reductase (LOC18032970) and P5CDH, delta-1-pyrroline-5-carboxylate dehydrogenase (LOC18045924) were identified. Both the loci coding for P5CS, PDH1 and P5CDH, were up-regulated in the CAR samples with a significantly higher expression, which was not observed in the MAC plants.

### Flavonoids and anthocyanins synthesis

A number of differentially expressed genes were related to the synthesis of anthocyanins like the anthocyanidin 3-O-glucosyltransferases (LOC18054166, LOC18047244), and flavonoids like chalcone-flavonone isomerase 3 (LOC18044429), chalcone synthases TRANSPARENT TESTA 4 (LOC18042808) and 7 (LOC18051925), flavonoid 3'-monooxygenase (LOC18050323), and flavonol synthase/flavanone 3-hydroxylase (LOC18037475). All them showed a consistently increased expression in the C15 samples and C30 vs. the M15 and M30 ones (Fig. 5b). We also identified a putative MYB111 gene LOC18031574 that’s has been shown to promote the synthesis of anthocyanins and flavonoids [64], that was up-regulated during cold treatment and was overexpressed in CAR samples.

### Hormone regulation

**Fig. 6:**
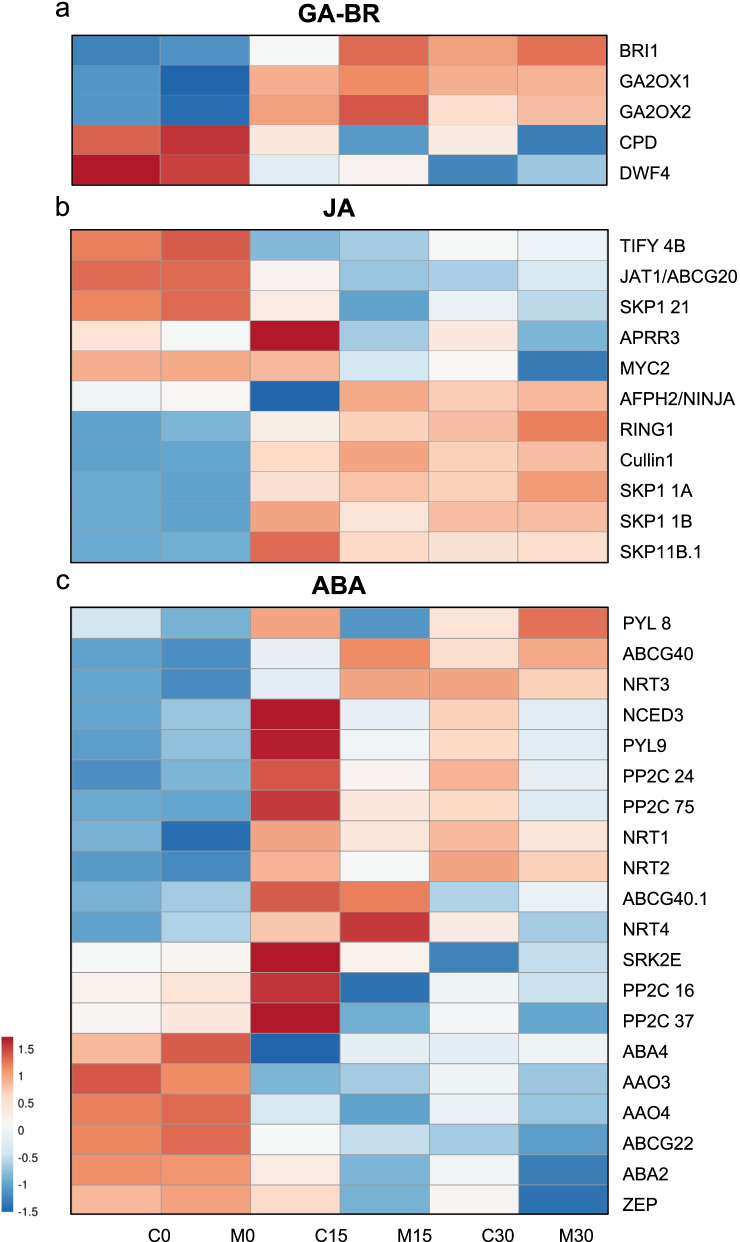
Heat map of the gene expression of hormones metabolism in Carrizo and *C. macrophylla* plants at 0, 15 and 30 cold days. **a** gibberellins- and brassionosteroid-related genes **b)** jasmonic acid-related genes and **c)** abscisic acid.related genes

Our analysis focused on the hormones that showed relevant differences in the 15-day samples: gibberellins (GA) and Brassinosteroids (BRs) (Fig. 6a), jasmonic acid (JA) (Fig. 6b) and abscisic acid (ABA) (Fig. 6c).

With respect GA, we found two differentially expressed loci coding for GA 2-beta-dioxygenase (GA2OX), LOC18050091) and LOC18049951, that were up-regulated in samples C15 and M15 in relation to C0 and M0 (Fig. 6a).

For Brassinosteroids, the orthologs of CONSTITUTIVE PHOTOMORPHOGENESIS AND DWARFISM, CPD (LOC18044077) and DWF4 (LOC18037059) were identified, which were down-regulated from 0 to 15 days, although expression levels were higher in the CAR samples, especially in DWF4. On the other hand, LOC18041991, coding for BRASSINOSTEROID INSENSITIVE 1-associated receptor kinase 1 protein (Brl1), was overexpressed at 15 days compared to 0 days, and in samples CAR vs. samples MAC (Fig. 6a).

Regarging JA signalling, different genes involved in the inactivation of the JAZ repressor proteins were up-regulated in both M15 and C15: Cullin1 (LOC18049048), RING1 (LOC18047608), and four SKP1 1 family members (LOC18037411, LOC18041740, LOC18033735, LOC18045891). Similarly, JA transporter JAT1/ABCG20 (LOC18039803) was activated in the 15-day samples. The repressor of jasmonate responses, TIFY 4B (LOC18047873), was down-regulated in C15 and M15, while AFPH2/NINJA (LOC18055945) showed no significant differences between 0 and 15 days, but the Carrizo samples had significantly fewer transcripts than the Macrophylla ones. Finally, the transcript levels of the key JA activator, bHLH transcription factor MYC2 (LOC18047048), remained high in CAR, while a significant reduction took place in M15 vs. M0 (Fig. 6b).

Finally, many genes involved in ABA synthesis and transport, as well as those coding for the receptors of the hormone, were differentially expressed in our samples (Fig. 6c).

Those genes involved in ABA biosynthesis, abscisic-aldehyde oxidase AAO3 (LOC18033897, LOC18034272), zeaxanthin epoxidase ZEP (LOC18036737), xanthoxin dehydrogenase ABA2 (LOC18038883) and neoxanthin synthase ABA4 (LOC18039410), were all down-regulated in M15 and C15 vs. M0 and C0, and only the expression of 9-cis-epoxycarotenoid dioxygenase NCED3 (LOC18046011) increased in the 15-day samples.

Several genes coding for ABA transporters belonging to the NRT1/ PTR and ABC G families, NRT1 (LOC18047775), NRT2 (LOC18047776), NRT3 (LOC18032684), NRT4 (LOC18031923O), ABCG40 (LOC18035930, LOC18036075), were up-regulated in the 15-day samples, except ABCG22 (LOC18038312), which was clearly down-regulated in the MAC samples and its expression levels remained high in the CAR ones.

A clearl expression increase was observed observed in C15 samples genes coding forABA receptors belonging to the PYL family (PYL 8/LOC18048127, PYL9/LOC18049819), as well as for ABA signaling integration, such as serine/threonine-protein kinase SRK2E (LOC18035057) or protein phosphatase 2C family members PP2C 16 (LOC18037418), PP2C 24 (LOC18034994), PP2C 37 (LOC18043434) and PP2C 75 (LOC18033189).

## Discussion

An RNA-Seq analysis was performed to investigate the genetic regulation of the response in citrus after exposure to low temperatures. Similar studies have been previously carried out in many species [8], including the citrus trifoliate orange *Poncirus trifoliata* [65]. However, our work provides novel approaches that can provide new insights into the process. The plants in our experiment were subject to long-term low temperature treatment with samples taken on days 0, 15 and 30, while most works base their results on short low temperature exposures lasting up to 72 h [65]. In this way, our results offer new data on the plant long-term response to cold by showing gene expression variation over a 30-day period.

We also measured the effects of low temperature on the commercial citrus variety sweet orange grafted onto two different rootstocks: Carrizo citrange, reported to be cold-tolerant, and *Citrus macrophylla,* one of the most sensitive to chilling citrus species. As we subjected the same scion to the same environmental conditions, the different obtained response could only be due to the different rootstock onto which it was grafted. Therefore, our analysis also provides relevant data about how rootstocks transfer their characters to scions by, in this case, providing resistance or sensitivity to low temperature.

### Physiological and transcriptomic data show relevant differences between sensitive and resistant rootstock samples

Our comprehensive comparative analysis of the physiological and transcriptomic changes that occurred in the sweet orange grafted onto a cold-sensitive citrus rootstock (MAC) and a resistant one (CAR) revealed marked differences between them. Our data indicate a possible correlation between changes in the accumulation of metabolites, hormone signaling and gene expression, caused by cold treatment, which might explain the sensitive and resistant phenotypes provided by rootstocks CAR and MAC.

The functional analysis of DEGs showed that all the previously described biological processes and pathways activated by cold response also worked in citrus. The biological process with the lowest FDR score was a significant response to cold, and other enriched terms and pathways included sugar metabolism, CBF regulon, light response, and hormonal regulatory pathways, all of which play essential roles in plants’ cold tolerance regulation.

Some enriched functions, such as response to chitin and Karrikin, which are related to defense against pathogens and are apparently unrelated to cold stress, have been recently associated with a response to biotic stresses [66, 67], which evidences the usefulness of enrichment analyses to reveal the new biological and molecular functions involved in a given biological process.

The large number of DEGs found in this RNA-seq study, as well as the many distinct biological processes in which they are involved, show the complexity of the response to low temperatures, which has been reported by many previous works [12, 68, 69]. Our results are similar to those obtained in an analogous study performed in trifoliate orange (*Poncirus trifoliata*). Both works reveal a large number of cold-responsive genes, including those encoding TFs, hormone signaling elements, and enzymes associated with the synthesis of protective metabolites [65]. The trifoliate orange transcriptome was analyzed after 72 h of cold treatment. In our experiment, samples were exposed to low temperature for 30 days. Thus our results indicate that the defense mechanisms shortly activated by cold stress, remain active after 1 month of cold exposure.

Most of the key genes involved in distinct cold response mechanisms were found to be differentially expressed in the 15D samples. However, relevant differences were found between both rootstocks. In Carrizo, 12691 DEGs were found in the C0-C15 comparison, but only 409 in the C15-C30 one, and none were relevant to cold adaption. The M0-M15 comparison yielded 14199 DEGs, while that of M15-M30 gave 4391, 10-fold the number of DEGs obtained for CAR and included many of the key regulatory genes involved in cold response like SOC1, APP3 and 7, LHY, ZAT11, ABA1 and 2, ABCG40, NRT4, PP2C 24 and 75, TIFY 4B, or MYC2. None of these regulatory genes displayed expression changes in C15 vs. C30. These data suggest that at 15 days, the Carrizo plants were fully adapted to low temperature exposure, while the Macrophylla ones kept struggling to do so with the expression of thousands of genes already adjusting to the adverse environment. Similar findings have been reported in *Prunus persica*, where major differences in the transcriptome were observed in peach chilling injury-sensitive and non-sensitive varieties, with quite a high degree of divergence between cultivars’ gene expression patterns [70]. Distinct expression profiles in response to cold have also found in potato when comparing the dynamic cold-response transcriptome of a sweetening resistant genotype to a sensitive one [71].

**Fig. 7:**
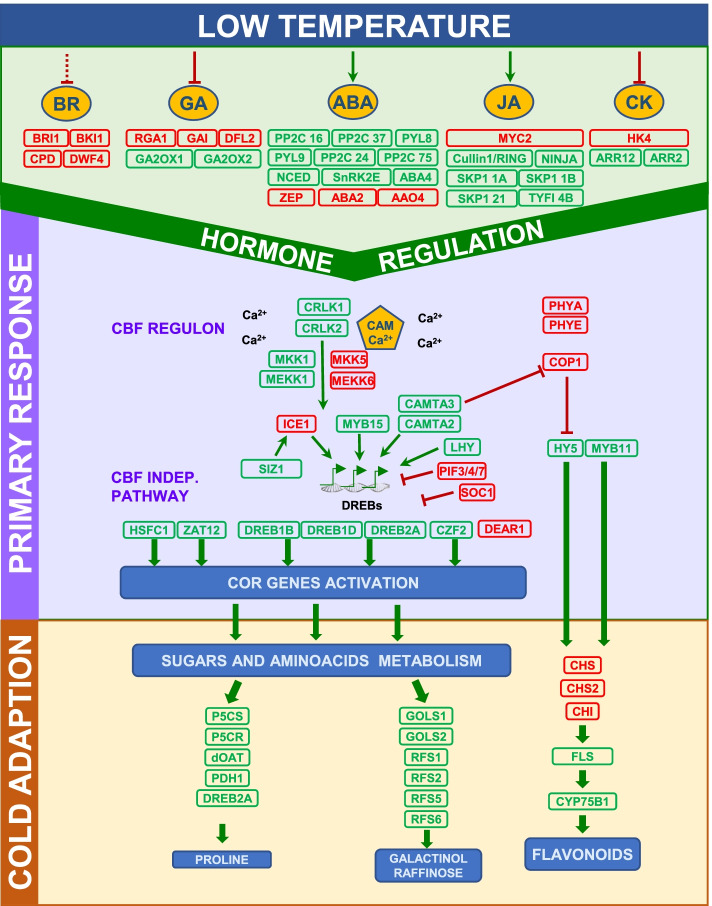
Summary figure including the most important DEGs and pathways that changed during long-term cold stress in citrus grafted plants. DEGs in green color represents genes that increased its expression and DEGs in red color represents genes that decreased its expression

In short, the overview of the physiological and transcriptomic data revealed the crucial regulatory pathways involved in response to low temperatures and abiotic stresses, which are discussed in the next sections. A summary of the differentially expressed relevant genes, as well as the pathways involved in the response to low temperature, are shown in Fig. 7.

### Accumulation of free sugars is higher in CAR and correlates with differences in gene expression

Considerable pieces of evidence demonstrate that sugar functions as an osmotic substance, a membrane stabilizer, and can be an antioxidant in cold stress responses [72]. The freezing tolerance level, and the accumulation of soluble sugars like sucrose, glucose and fructose, parallel one another in different photoperiods during cold acclimation. This scenario indicates that sugar accumulation is a fundamental component of enhanced freezing tolerance [73].

Our analyses showed a clear and continuous increase in the total soluble sugars in the resistant Carrizo samples (Fig. 1), which was not so evident in the sensitive Macrophylla ones. The raffinose concentration increased 336-fold in the CAR plants and 299-fold in the MAC plants, which was by far the largest increase. The raffinose family of oligosaccharides has been found to accumulate in different abiotic stresses like drought or cold [74], and galactinol and raffinose levels correlate with increased tolerance to salinity and chilling in paraquat [75]. Long-term cold stress considerably increases galactinol and raffinose contents in rice [76]. Raffinose sugar is found in the chloroplast [77, 78] and seems to play an important role by stabilizing photosystem II in *A. thaliana* under freezing conditions [41]. Raffinose synthesis alone is not sufficient for inducing cold tolerance or cold acclimation in *A. thaliana,* but plants are much better protected from oxidative damage when the raffinose concentration rises [79].

This sharp increment in the raffinose concentration correlates with the significantly increased expression of galactinol synthase and raffinose synthase genes, both involved in raffinose biosynthesis. Similar results and an increase in the transcript levels of the genes related to galactinol and raffinose synthesis preceding the accumulation of raffinose have been detected and found in rice [76] and Arabidopsis [80].

Therefore, the greater accumulation of free sugars, specially raffinose and fructose, in resistant CAR plants would increase adaption to low temperatures, while the sensitive MAC ones would be more exposed to damage caused by cold.

Accumulation of soluble sugars is usually accompanied by starch degradation in many plant species, including grasses, such as barley, or woody plants like lychee tree or poplar [81]. However, in our study, the starch concentration did not lower in the MAC plants, and even increased in the CAR samples, which correlates with the down-regulation of the expression of the citrus β-amylase gene (LOC18048130) observed in all the samples. These results contrast with those obtained in trifoliate orange which, under cold stress, showed considerably more PtrBAM3BAM activity and greater accumulation of maltose and soluble sugars. These data suggest that β-amylase is a member of β-CBF regulon in *P. trifoliata*a*,* and plays an important role in cold tolerance by modulating the levels of soluble sugars [53]. Nevertheless, several studies have also reported increased starch accumulation in plants under cold stress. In tomato (*Solanum lycopersicum*), cold-activated starch accumulation in sensitive tomato species and starch content in leaves were 4- to 5-fold higher in cold-sensitive *Solanum spp*., but remained unchanged in three cold-adapted ones [82]. Furthermore, sugar levels and photosynthesis did not change in tolerant species, but either increased or decreased in susceptible ones [83]. Similarly, cold treatment in *A. thaliana* increased both starch content and the accumulation of the major starch-degrading product, maltose [45]. The reasons for these discrepancies remain unclear and the response might depend on the exact experimental conditions, the involved haplotypes, etc. [81]. Therefore, obtaining a more consistent view of changes in starch metabolism during cold stress in citrus would require running experiments under similar conditions to make direct comparisons between species and different cold treatments.

### Proline accumulation

Free proline accumulation as a plant response to abiotic stresses has been repeatedly reported, with a direct relation existing between proline accumulation and plant stress tolerance [84]. Our results agree with this observation as significant proline accumulation was found in our samples during cold treatment. Furthermore, a differential 2-fold proline accumulation was observed in the resistant CAR plants vs. the sensitive MAC ones. Likewise, the genes involved in proline synthesis, such as *P5CS*, *PDH1*, and *P5CDH,* displayed a significantly increased expression in the CAR samples compared to the MAC ones. Our data corroborate the results obtained in a long-term cold stress experiment performed in Carrizo citrange, which reported an increment in both proline concentration and proline synthesis gene expression levels [58].

An evaluation of proline levels during the low temperature acclimation of several *Citrus* species has shown that accumulated free proline was 3- to 6-fold higher in cold acclimated than in non-acclimated tissues, and higher proline significantly correlated with plants’ ability to survive freeze damage [85]. More recently, studies into cold stress tolerance in chickpea and pine tree*s* compared cold-tolerant and cold-sensitive cultivars to show an increase in proline in leaves exposed to cold stress in both species [86, 87].

Hence we evidence a differential accumulation of total free sugars and proline in the CAR plants, which would contribute to protect them from cold damage.

### Gene regulation of the cold response

Our RNA-seq study identified most of the key regulatory factors of cold response in plants. As expected, the genes involved in the MAP kinase, light, CBF and hormone signaling pathways were differentially expressed from 0 to 15 days, which indicates that plants had adapted to low temperature exposure. These pathways have been extensively analyzed in many species, including trifoliate orange [65], and our results very much agree with these previous results. Thus we focused the discussion on the differences between the CAR and MAC samples to gain some insight into the origin of cold sensitiveness or hardiness in citrus species.

Intraspecies comparisons C0/C15 and M0/M15 evidenced that most of the genes acting as promoters of cold adaption were up-regulated, while those acting as repressors were down-regulated in the 15-day samples vs. the 0-day ones. Alternatively, the interspecies expression analysis, M15/C15, showed that a relevant number of the genes that acted as promoters displayed significantly higher expression levels in C15 in relation to M15, and, on the contrary, those genes repressing cold adaption had lower expression levels. The final expression balance would favor cold acclimation in the resistant Carrizo plants, which would diminish in the sensitive Macrophylla ones.

There are many pieces of evidence for the critical role of CBF transcription factors in cold acclimation [25]. So the up-regulation in all the 15D samples of the three *CBF* citrus orthologs, *DREB1B*, *DREB1D* and *DREB2A*, was not unexpected. Remarkably, the FC and transcript levels were much higher in the C15 than in the M15 samples. It has been reported that the up-regulation of *CBF* genes enhances cold hardiness in apple [31], barley [32], potato [30] and poplar [27]. These results could coincide with ours as the up-regulation of the *CBF* genes in Carrizo vs. Macrophylla would correlate with the hardiness or sensitivity displayed by each rootstock.

Transcription factor *ICE1* regulates the expression of *CBF* genes under cold conditions, is expressed constitutively in Arabidopsis, and its overexpression in wild-type plants enhances the expression of the CBF regulon and improves freezing tolerance [13]. The citrus *ICE1* gene that we identified, LOC18051975, was identical to *PtrbHLH* from *Poncirus trifoliata* [55]. The expression pattern of *PtrbHLH* under cold stress reached the highest level at 48 h, with a drop at 72 h which could not explain its severe expression reduction in the 15-day samples vs. controls. Further research is required with samples taken at shorter intervals to understand the dynamics of IC1 expression during long-term cold stress.

However, ICE1 activity regulation takes place at the protein level, and ICE1 levels are modulated by ubiquitylation, which leads to its degradation, while sumoylation stabilizes ICE1 by preventing ubiquitylation [16, 68]. Thus it was interesting to find that citrus *SlZ1*, coding for an E3 SUMO-protein ligase, was overexpressed in the 15-day and 30-day samples and, once again, its expression level was much higher in the C15 samples than in the M15 ones. Higher levels of SlZ1 transcripts would promote ICE1 protein stabilization, which would lead to the up-regulation of *CBF* genes in Carrizo plants.

Calmodulin-binding transcription activator (CAMTA) transcription factors are positive regulators of *CBF*s, of which CAMTA2 and CAMTA 3 have been reported to directly bind to the *CBF2* promoter [15, 88]. Once again, citrus genes LOC18034255 and LOC18031678, respectively coding for CAMTA2 and CAMTA 3, were more up-regulated in the 15-day samples, although no significant differences could be found for *CAMTA3* between C15 and M15, and the expression level for *CAMTA2* was even higher in M15.

Cold-induced CBF expression can be affected by light quality, the circadian clock and photoperiod. It is known that the late elongated hypocotyl (LHY) protein directly binds to CBF promoters and positively regulates CBF expression [36]. In our experiment, the expression levels of the citrus *LHY* gene (LOC18047565) were always much higher in the CAR samples than in the MAC ones (4716,40 TMMs in C15 vs. 1445,42 TMMs in M15). Higher LHY levels in Carrizo would lead, as seen before, to a higher up-regulation of *CBF* genes to improve cold resistance in this rootstock.

In addition to their roles in plant photomorphogenesis, cross-talk between phytochrome-mediated light signals and cold signaling pathways has been identified in Arabidopsis and tomato [89, 90]. *PHYA* and *PHYE* play antagonistic roles, and they positively and negatively regulate cold tolerance, respectively [90, 91]. Citrus loci *PHYA*/LOC18035337 and *PHYE*/LOC18053186 display expression patterns which agree with their roles, thus *PHYA* is up-regulated and *PHYE* is down-regulated during cold treatment. As we describe before, *PHYA* levels were significantly higher in the CAR samples, which would once again favor enhanced cold acclimation in Carrizo plants. On the contrary, the *PHYE* expression level was higher in the MAC samples, which would produce a stronger repression of cold acclimation and would contribute to the cold-sensitive phenotype of Macrophylla plants.

Mitogen-activated protein kinase cascades play a relevant role in plants’ cold adaption, and typically comprises three protein kinases, MEKK, MKK, and MPK, which serially act [12]. The MKK4/5–MPK3/6 pathway promotes ICE1 degradation and the repression of *CBF* genes*.* In our study, the citrus gene coding for *MKK5*, LOC112100736, was down-regulated, and the expression levels in the MAC samples were at least 2-fold those found in the CAR ones. Hence in Macrophylla plants, greater MKK5 activity would lower ICE1 levels to cause more marked *CBF* genes repression, which would agree with Macrophylla cold sensitivity.

The citrus HY5 gene, LOC18054790, showed a slightly increased expression in the C15 samples, but remained unchanged in M15. However, the amount of the HY5 transcripts was 4-fold higher in the C15 samples compared to M15, and almost 3-fold in C30 in relation to M30. bZIP transcription factor HY5 is a hub of both cold response and photomorphogenesis that positively regulates cold-induced gene expression to ensure complete cold acclimation development. HY5 mediates plant responses, such as anthocyanin biosynthesis or hormonal responses by ABA, GAs, cytokinin (CK) and auxins [92, 93]. The role of HY5 in cold acclimation is crucial, as shown in *A. thaliana*, for controlling the induction of nearly 10% of all cold-inducible genes [94]. Our results suggest that the up-regulated *HY5* expression would boost the cold adaption process in Carrizo plants, while its down-regulation would make the response to low temperatures in Macrophylla plants difficult.

Cold response induces the synthesis of flavonoids and anthocyanins, that enhance cold acclimation [64, 95, 96]. Anthocyanins are photoprotective agents that shade and protect the photosynthetic apparatus by absorbing excess visible UV light and scavenging radicals in different abiotic stresses [97, 98]. For example, rich anthocyanins red pear fruit (cv. Anjou) and purple pepper leaves (cv. Huai Zi) show stabler PS II photosynthetic capacity and greater photo-oxidation tolerance compared to non anthocyanin tissues [97, 99, 100]. Our results indicate that the biosynthesis of anthocyanin and flavonoids would be significantly more active in Carrizo, which would be able to reduce ROS damage more efficiently than Macrophylla, where ROS damage would be more difficult to handle, which could be another possible cause of the Macrophylla cold-sensitive phenotype.

### Hormonal regulation and rootstock/scion interactions

Plant hormones function as governors of signal events in cold stress responses by displaying synergistic or antagonistic effects on the biosynthesis and signaling outputs of other hormones to create a complex network of hormonal interactions [101]. A transcriptomic study in Arabidopsis has shown that cold and light can induce the expression of the genes related to ABA biosynthesis to prepare plants for cold acclimation [102]. In tomato, it has been proposed that the *PHYA* level increases at cold temperatures, which results in the up-regulation of *HY5* [103]. HY5 promotes ABA biosynthesis and GA catabolism by generating a low GA/ABA ratio, capable of stopping plant growth and promoting cold tolerance [103].

Growth-promoting hormone gibberellin (GA) is targeted by cold stress, which results in a reduction in bioactive GA that suppresses growth and late flowering [104]. Increased expressions of citrus genes *GA2OX 1*/LOC18050091 and *GA2OX 2*/LOC18049951 during our experiment would lower GA levels by causing plant growth arrest and promoting cold adaption. However, no significant differences were found between samples CAR and MAC.

Brassinosteroids also promote plant growth but, unlike GA, seem to positively control cold stress responses[105]. Our results show an overexpression of *BrI1*, *CPD* and *DWF4* in the C15 samples, that would significantly increase BRs synthesis in Carrizo, promoting cold adaption and contributing to its chilling resistance phenotype.

Jasmonic acid is an oxylipin whose levels increase under cold stress in different plant species like rice and Arabidopsis, which correlates with a higher expression of JA biosynthetic genes [106, 107]. The up-regulation of the genes involved in JA signaling, such as *Cullin1*, *RING1*, *SKP1 1* family and *MYC2*, together with the down-regulation of *AFPH2/NINJA* and *TIFY 4B*, repressors of JA signaling, suggests the relevant role of JA in cold adaption in our experiment. However, the gene expression data did not correlate with the JA quantification in leaves and roots, and the amount of JA observed in the C15 and M15 samples lowered. Further research must be performed to find the reason for the uncoupling of gene expression and JA accumulation in this experiment.

Abscisic acid is a central regulator of cold stress signaling with emerging roles in the CBF dependent pathway. In many plant species, higher ABA levels that correlate with increased ABA biosynthesis take place in response to cold, and ABA mutants display altered cold resistance [101]. ABA acts as a long-distance transporter signal that mediates root-to-shoot communication under stress conditions [76]. In our study, many of the genes involved in ABA synthesis and transport, and those coding for the receptors of this hormone, were differentially expressed. The genes involved in ABA biosynthesis were down-regulated in M15 and C15 compared to M0 and C0, which indicates that ABA production in leaves decreased. Previous reports state that cold stress in Arabidopsis modifies ABA biosynthesis and catabolism, and also affects ABA transport and homeostasis [108].

The down-regulation of the genes involved in ABA biosynthesis in the M15 samples correlated with the lower ABA concentration in Macrophylla plant leaves. However, ABA concentration remained constant in samples C15 despite the down-regulation of the genes involved in ABA biosynthesis that also took place. One possible reason for this result could be the rise in ABA concentration observed in Carrizo plant roots, which was significantly higher at 15 and 30 cold days than in the MAC plants, where no increase was observed (Fig. 2). The ABA quantification results would agree with the transcript expression patterns: genes coding for the NRT1/PTR and ABCG families of ABA transporters were up-regulated in the 15-day samples. Moreover, the expression of the genes coding for the ABA receptors PYL family, and for ABA signaling integration, like SRK2 E kinase or the protein phosphatase PP2C family, clearly increased in the C15 samples, with significantly higher normalized expression values (TMMs) in C15 than in M15. These data suggest that the large amount of ABA produced in Carrizo roots could be transported to leaves so that its concentration would remain constant, and would allow the activation and promotion of the ABA signaling response to cold in CAR plants during the 30-day low temperature treatment. This would not happen in Macrophylla because no increase in ABA concentration took place. Hence lack of ABA signaling would lead to a cold sensitive phenotype.

These data suggest that the different sensitivity to low temperatures shown by the sweet orange scion could be transferred by the respective resistant or sensitive rootstock onto which it was grafted. The influence of rootstocks on many scion biology aspects is well-established, and molecular aspects of root-to-shoot and/or shoot-to-root signaling events are starting to be known and show how grafting triggers differential responses between the scion and rootstock [109, 110]. Grafting is widespread to improve plant performance in terms of yield, quality and resilience to abiotic and biotic stresses, [111]. The use of tolerant rootstocks to different abiotic stresses, such as drought, salinity, drastically rising or decreasing temperature, etc., is becoming indispensable in this global climate change era [112, 113]. Highly tolerant rootstocks have been shown to improve cold tolerance in eggplant, tomato and cucumber [114–116].

In citrus, a tetraploid Carrizo citrange rootstock enhances the natural chilling stress tolerance of the common clementine, *C. clementina*, which reveals that the ability of Carrizo citrange to promote cold tolerance does not depend on the species it is grafted onto as we obtained similar results in our experiments with sweet orange, *C. sinensis,* employed as the scion [51].

In another study, the impact of grafting on the cold responsive gene expression in Satsuma mandarin, *Citrus unshiu*, has been addressed by real-time RT–PCR following exposure to cold shock or cold acclimation treatments. *P. trifoliate*, one of the Carrizo Citrange parentals, was used as a rootstock. The Poncirus rootstock significantly affected the gene expression in the *C. unshiu* scion, and major expression changes were detected during cold stress in different species, which would agree with the results that we obtained with the whole transcriptome in sweet orange [117].

## Conclusions

This work shows the results of studying the long-term effect of cold exposure on citrus plants. We provide metabolomic and transcriptome data, which reveal how the response mechanisms activated by cold remain active after 30 days of low temperature exposure.

By analyzing the transcriptomic changes in samples from a citrus commercial variety, sweet orange, grafted onto two rootstocks with different sensitivities to low temperatures, we demonstrate the extent to which rootstocks are able to modify the scion response to abiotic stress. The obtained results allowed us to propose a mechanism by which the resistant rootstock might promote cold hardiness in the scion via ABA signaling, which would up-regulate the regulatory pathways involved in cold adaptation.

However, more research will be required to confirm the role of ABA signaling in cold tolerance in citrus, as well as to describe in detail the scion-rootstock interactions that lead to the different responses to cold in the scion.

## Methods

### Plant material and growth conditions

Eighteen-month-old plants of the delta seedless variety grafted onto Carrizo citrange [*C. sinensis*(L.) Osbeck. x *Poncirus trifoliata* (L.) Raf.] and *Citrus Macrophylla* were obtained from a nursery (Viveros Sevilla SA, Sevilla/Tocina road km 147, 41310, Brenes, Spain) and no permission was necessary to collect the plants. Plants were grown individually in 4-liter opaque plastic pots filled with substrate composed of peat, coconut fiber, sand and perlite (40:25:25:10). Plants were irrigated twice weekly with the following basal nutrient solution (pH 6.0) at half strength: 5 mM Ca(NO_3_)_2_, 1.4 mM KNO_3_, 2 mM MgSO_4_, 0.6 mM H_3_PO_4_, 20 µM Fe-EDDHA, 7.6 µM ZnSO_4_·7H_2_O, 0.50 µM CuSO_4_·5H_2_O, 50 µM H_3_BO_3_, 0.50 µM MoO_3_, 54 µM MnSO_4_·H_2_O. Plants were acclimated for 2 weeks before the experiments began under greenhouse conditions (26-28/16-18°C, 70-80% and a 16-hour photoperiod).

Plants were selected according to their size uniformity. The control group comprised six plants, three for the CAR genotype and three for MAC. It was left under controlled greenhouse conditions, which were the same conditions before experiments began: 26-28/16-18°C temperature, 70-80% humidity and a 16-hour photoperiod (16 h light/8 h dark). The cold-treated plants were formed by 12 plants: three for CAR at 15 days, three for MAC at 15 days, three for CAR at 30 days and three for MAC genotype at 30 cold days. The cold plants were cultured in a Versatile Environmental Test Chamber (MLR-350, Sanyo) with a temperature range from 1ºC to 2ºC, both day and night, and a 16-hour photoperiod and 8 h of darkness (500 μmol m−2 s−1, 400-700 nm). Relative humidity was maintained at approximately 80%. Plants were irrigated once weekly with the afore-described nutrient solution (Additional File 3).

### Soluble sugars and starch quantification

Soluble sugars and starch were measured biweekly in the lyophilized and milled leaves (100 mg DW). Soluble sugars and starch were analyzed by a colorimetric method based on [118]. Samples were mixed with heated ethanol and centrifuged. The liquid part contained sugars and the precipitate contained starch. For the sugars and starch determinations, anthrone-acid solution was added and samples were placed in a boiling water bath. The results were read at 630 nm (Lambda 25, PerkinElmer, Shelton, CT, USA).

### Proline quantification

The free proline concentration in leaves was determined according to Hu, Delauney and Verna [119]. Samples were collected at 0, 1, 2, 4, 7, 15 and 30 days, and frozen and lyophilized. Then 250 mg were weighed and homogenized (Vortex) in 1.5 mL of sulfosalicylic acid (3%) for 1 min, centrifuged at 14000 rpm for 5 min (Eppendorf Centrifuge 5810R, AG, Hamburg, Germany) and the supernatant was stored at 4°C. An aliquot (0.2 mL) was incubated with 0.5 mL of sulfosalicylic acid (3%), 0.7 mL of reactive ninhydrin acid reagent (ninhydrin, phosphoric acid 6 M, glacial acetic acid 60%) and 0.6 mL of glacial acetic acid (99%) in a dry bath at 100°C for 1 h (Thermostatic Bath BD, Bunsen SA, Humanes, Spain). Samples were cooled in an ice bath for 15 min and absorbance was measured at 520 nm (Lambda 25, PerkinElmer, Shelton, CT, USA).

### Primary metabolites

The primary metabolite analysis was performed at the Instituto de Biología Molecular y Celular de Plantas (UPV-CSIC, Valencia, Spain) on the Metabolomics Platform by a modified method of that described by Roessner et al [120]: 100 mg of leaves per sample were homogenized with liquid nitrogen and extracted in 1400 µL of 100% methanol supplemented with 60 µL of internal standard (0.2 mg ribitol in 1 mL of water) for 15 min at 70°C. The extract was centrifuged for 10 minutes at 14000 rpms. The supernatant was transferred to a glass vial and 750 µL of ChCl_3_ and 1500 µl of water were added. The mixture was vortexed for 15 sec and centrifuged for 15 min at 14000 rpms. Then 150-µL aliquots of the methanol/water supernatant upper phase were dried in a vacuum for 6–16 h.

For derivatization purpose, the dry residues were dissolved in 40 µL of 20 mg/mL of methoxyamine hydrochloride in pyridine and incubated for 90 min at 37ºC, followed by the addition of 70 µL of MSTFA (N-methyl-N-[trimethylsilyl]trifluoroacetamide) and 6 µL of a retention time standard mixture (3.7% [w/v] mix of fatty acid methyl esters ranging from 8ºC to 24ºC) with further incubation for 30 min at 37ºC.

Sample volumes (2 µL) were injected in the split and splitless modes to increase the metabolite detection range in a 6890 N gas chromatograph (Agilent Technologies Inc. Santa Clara, CA, USA) coupled to a Pegasus 4D TOF mass spectrometer (LECO, St. Joseph, MI, USA). Gas chromatography was performed in a BPX35 (30 m × 0.32 mm × 0.25 μm) column (SGE Analytical Science Pty Ltd., Australia) with helium as the carrier gas at constant flow: 2 ml/min. The liner was set at 230ºC. The oven program was 85ºC for 2 min, 8ºC/min ramp until 360ºC. Mass spectra were collected at 6.25 spectra s−L within the 35–900 m/z range and ionization energy of 70 eV. Chromatograms and mass spectra were evaluated by the CHROMATOF program (LECO, St. Joseph, MI, USA).

### Hormone quantification

The quantification of ABA and JA has been carried out by Seo, Jikumaru and Kamiya [121] following the protocol quantification in lyophilized and milled leaves at 0, 15 and 30 cold days. In short, the material (about 100 mg of dry leaves and roots) from 0, 15 and 30 cold days was suspended in 80% methanol-1% acetic acid containing internal standards, and was mixed by shaking for 1 h at 4ºC. The extract was kept at -20ºC overnight and then centrifuged. The supernatant was dried in a vacuum evaporator. The dry residue was dissolved in 1% acetic acid and passed through an Oasis HLB (reverse phase) [121].

For the ABA and JA quantifications, the dried eluate was dissolved in 5% acetonitrile-1% acetic acid, and hormones were separated using an autosampler and reverse phase UHPLC chromatography (2.6 µm Accucore RP-MS column, 50 mm length x 2.1 mm i.d.; Thermo Fisher Scientific) with a 5-50% acetonitrile gradient containing 0.05% acetic acid at 400 µL/min for 14 min.

Hormones were analyzed in a Q-Exactive mass spectrometer (Orbitrap detector; Thermo Fisher Scientific) by targeted Selected Ion Monitoring (SIM). The concentrations of hormones in extracts were determined using embedded calibration curves and the Xcalibur 4.0 and TraceFinder 4.1 SP1 programs. The internal standard for the ABA and JA plant hormone quantification was the deuterium-labeled hormone (^2^H6-ABA) and dhJA, respectively.

### Statistical analyses

For the statistical analyses, all the resulting values were the mean of six independent plants per treatment. The RT-PCR values were the mean and standard deviation of three biological replicates and three technical replicates per plant. Data were submitted to an analysis of variance (multifactor ANOVA) using Statgraphics Centurion, version 16.1 (Statistical Graphics, Englewood Cliffs, NJ, USA) prior to testing for normality and homogeneity. When the ANOVA showed a statistical effect, means were separated by least significant differences (LSD) at *P* < 0.05.

### RNA isolation

Leaf samples were obtained biweekly, collected in liquid N_2_ and stored at -80ºC. Total RNA was isolated from 100 mg of plant tissue using the RNeasy Plant Mini Kit (Qiagen) with RLT-β-mercaptoethanol (Sigma-Aldrich) buffer. Contaminant genomic DNA was removed with the RNase-Free DNase Set (Qiagen, CA, USA) by on-column digestion according to the manufacturer’s instructions. Purified RNA (2 µg) was reverse-transcribed with SuperScript® III Reverse Transcriptase (RT) (Life Technologies, Carlsbad, CA, USA) in a total volume of 10 μL. First-strand cDNA was 50-fold diluted and 2 μL were used as a template for the quantitative real-time RT-PCR in a final volume of 20 μL. Quantitative real-time PCR was performed in a StepOnePlus Real-Time PCR System (Life Technologies, Carlsbad, CA, USA) using TB Greenpremix Ex Taq (TliRNaseH plus) (Takara Europe, S.A.S, Saint Germain en Laye, France). The PCR protocol consisted of 10 min at 95ºC, followed by 40 cycles of 15 sec at 95ºC, and 1 min at 60ºC. The specificity of the reaction was assessed by the presence of a single peak on the dissociation curve and through the size estimations of the amplified product by agarose electrophoresis. All the specific primers (Table 3) were tested before PCR reaction and an efficiency=2 was obtained [122]. The CiclevActin and CiclevUBC4 transcripts, amplified with specific primers, were used as reference genes [123, 124], and a single-factor ANOVA and linear regression analyses of the CT values were performed to examine the variation in our reference genes [125]. The normalization factor of the reference genes was calculated by the geometric mean of the values of both genes [125]. The relative expression was measured by the relative standard curve procedure with 5 points of dilutions [122]. The results were the average of three independent biological replicates with three technical replicates per biological sample.

**Table 3:** List of the primers used for quantitative real-time PCR

NCBI Code^a^	Gene Name	Ciclev Gene^b^	Primers
LOC18033409	*DREB1D*	Ciclev10007068	FOR 5' TGGGATGCCCAGATTGTTG 3' REV 5' CCTCCACAATTAGACTGAGGTGGT 3'
LOC18049462	*DREB1B*	Ciclev10021923	FOR 5' AGACCTCGTGATGATGAACTTGA 3' REV 5' GTGTTTTCTTGCATCGTTTTCTGT 3’
LOC18035610	*WCOR413*	Ciclev10029293	FOR 5' TCTTCCCAAGACATTTCCCAG 3' REV 5' CAGCAAATAACAAGCGATGGCA 3’
LOC18039477	*PDH*	Ciclev10011584	FOR 5' ATCTGCCAAGTCTCTGCCTC 3' REV 5' GCTTCCACGGGAGATTAAATGA 3'
LOC18044634	*P5CS*	Ciclev10030839	FOR 5' CCCTTGGGTGTTCTCTTGATTG 3' REV 5' CTTGTGCAGAATTGCATTTGA 3'
LOC18045924	*P5CDH*	Ciclev10019562	FOR 5' ATCACCTTGGACAGCAGAGC 3' REV 5' CCATATAGAGCGCACCCATCA 3'
LOC18046011	*NCED*	Ciclev10019364	FOR 5'TGATGGCTACATTCTCGCTTTTGTA 3' REV 5' GCTTATGAACGTCCCGTGGA 3'
LOC18043434	*PP2C*	Ciclev10031682	FOR 5' CTCTCGTGCTGTGCTCTG 3' REV 5' CGGCGGCTTCGATTCTAAG 3’
ATUBC4	*UBC4*	Ciclev10009771	FOR 5’ TGGACGCTTCAGTCTGTTTG 3’ REV 5’ TCGTCAATCACCCCTTCTTT 3’
β-ACTIN	*ACTIN*	Ciclev10025866	FOR 5' CAGTGTTTGGATTGGAGGATCA 3' REV 5' TCGCCCTTTGAGATCCACAT 3'

^a^ Code refers to the transcript name in the NCBI database available at (https://www.ncbi.nlm.nih.gov/nucleotide/)

^b^ Code refers to the transcript name in the database available in the International Citrus Genome Consortium (https://phytozome.jgi.doe.gov/pz/portal.html)

### RNA sequencing library preparation and sequencing

Total RNA from leaves was used for library construction. The Poly(A)+ mRNA fraction was isolated from total RNA and the cDNA libraries were obtained following Illumina´s recommendations. Briefly, poly(A)+ RNA was isolated on poly-T oligo-attached magnetic beads and chemically fragmented prior to reverse transcription and cDNA generation. The cDNA fragments then underwent an end repair process, a single ‘A’ base was added to the 3’ end, followed by the ligation of adapters. Finally, products were purified and enriched with PCR to create the indexed final double-stranded cDNA library.

The quality of libraries was analyzed by a Bioanalyzer 2100, a High Sensitivity assay. The quantity of libraries was determined by real-time PCR in a LightCycler 480 (Roche). The pool of libraries was sequenced by paired-end sequencing (100 x 2) in an Illumina HiSeq 2500 sequencer.

### RNA-Seq and differential expression analyses

RNA-Seq fastq files were pre-processed with Trimmomatic 0.38 [126], and the reads with an average quality smaller than 25 and shorter than 36 bases were filtered as implemented in the OmicsBox suit. The transcriptome of what was obtained was used as a reference for mapping.

The transcript-level quantification tool was estimated from the RNA-Seq reads (FASTQ) using the RSEM software package, which allocates multimapping reads among transcripts by an expectation maximization approach [127] based on a fast gapped-read alignment with Bowtie 2 [128]. The reference transcript sequences were obtained from the NCBI *Citrus clementina* Annotation Release 100 (GCF_000493195.1).

Read counts were normalized with the Trimmed mean of the M values (TMM) method [129]. Pairwise differential expression analyses were carried out with the EdgeR package 3.28.0 using the GLM (Quasi Likeli hood F-Test) with 0.05 taken as the cut-off value [130].

Gene set Enrichment of GO terms for the differentially expressed gene was carried out using FatiGO, which employs Fisher's Exact Test for the statistical assessment of any annotation differences between two sets of sequences.

Expression heat maps were constructed with ClustVis. The original TMM values were ln(x)-transformed, and rows were clustered using correlation distance and average linkage [131].

Raw data are deposited in the European Nucleotide Archive, ENA (EMBL-EBI) with Project Number PRJEB20758 (https://www.ebi.ac.uk/ena/data/view/PRJEB20758) (Additional File 4).

## Supplementary Information

**Additional file 1:Additional Figure 1.** RNA-seq transcript validation of DEGs. Bars diagram indicates a relative TMMs from RNA-seq analysis and red line was the relative transcript gene level from RT-PCR. Figure a) DREB1D (LOC18033409), b) DREB1B (LOC18049462), c) PDH (LOC18039477), d) WCOR413 (LOC18035610), e) P5CS (LOC18044634), f) P5CDH (LOC18045924), g) NCED (LOC18046011) and h) PP2C (LOC18043434). Samples were measured in the leaves of the CAR and MAC plants grafted under cold (1°C) and control conditions for 0, 15 and 30 days. The values are the means±SE of three biological replicates (*n*=3) and three technical replicates per biological sample. The treatment effect tested by multi-way ANOVA, different letters indicate significant differences (*P* < 0.05) according LSD.

**Additional file 2:Additional Table 1.** Most represented KEGG pathways. KEGG is developed by Kanehisa Laboratories from https://www.kegg.jp/kegg/.

**Additional file 3:Additional Figure 2.** Explanatory diagram of experimental procedure.

**Additional file 4:Additional Table 2.** Raw data lectures in European Nucleotide Archive.

## Data Availability

RNA-seq raw data are deposited in the European Nucleotide Archive, ENA (EMBL-EBI) with Project Number PRJEB20758 (https://www.ebi.ac.uk/ena/data/view/PRJEB20758).

## Abbreviations

CAR: Carrizo citrange
MAC: *Citrus macrophylla*
DEGs: Differentially expressed genes
GO: Gene ontology
KEGG: Kyoto encyclopedia of genes and genomes
ABA: Abscisic acid
COR: Cold-responsive
CBF1: C-repeat/DRE binding factor 1
CBF2: C repeat/DRE binding factor 2
CBF3: C-repeat/DRE binding factor 3
ICE1: Inducer of CBF expression 1
SIZ1: Salt-induced zinc finger protein1
HOS1: High expression of osmotically responsive genes 1
MPK: Mitogen-activated protein kinase
MKK: Mitogen-activated protein kinase kinase
MEKK: Mitogen-activated protein kinase kinase kinase
LHY: Late elongated hypocotyl
CAMTA: Calmodulin-binding protein
HY5: elongated hypocotyl 5
MYB15: MYB domain protein 15
HSFC1: Heat shock transcription factor C1
ZAT12: Zing finger responsive to high light 41
CZF1: Salt-inducible zinc finger 2
COR14b: Cold-responsive 14b
ZT8: Zeitgeber time 8
ZT20: Zeitgeber time 20
ZT4: Zeitgeber time 4
ZT16: Zeitgeber time 16
PS II: Photosystem II
CTV: Citrus tristeza virus
GABA: Gamma-aminobutyric acid
JA: Jasmonic acid
DREB1B: Dehydration-response element-binding protein 1B
DREB1D: Dehydration-response element-binding protein 1D
DREB2A: Dehydration-response element-binding protein 1A
COP1: Constitutive photomorphogenic 1
ZAT11: Zinc finger protein 11
DEAR1A: DREB and EAR motif protein 1A
PHY A: Phytochrome A
PHY B: Phytochrome B
PIF3: Phytochrome interacting factor 3
PIF4: Phytochrome interacting factor 4
PIF7: Phytochrome interacting factor 7
CRLK1: Calcium/calmodulin-regulated receptor-like kinase1
CRLK2: Calcium/calmodulin-regulated receptor-like kinase2
GOLS1: Galactinol synthase 1
GOLS2: Galactinol synthase 2
RFS1: Raffinose synthase
RFS2: Raffinose synthase
RFS5: Raffinose synthase
RFS6: Raffinose synthase
FBA: Fructose-1,6-bisphosphate aldolase
BAM1: Beta-amylase 1
dOAT: Ornithine aminotransferase
P5CS: delta-1-pyrroline-5-carboxylate synthase
PDH1: Proline dehydrogenase 2
P5CR: Pyrroline-5-carboxylate reductase
P5CDH: delta-1-pyrroline-5-carboxylate dehydrogenase
GA: Gibberellin acid
BRs: Brassinosteroids
GA2OX: Gibberellin 2-beta-dioxygenase
CPD: Constitutive photomorphogenesis and dwarfism
DWF4: Dwarf 4
BRI1: Brassinosteroid insensitive 1
SKP1: S phase kinase-associated protein 1
JAT1/ABCG20: Jasmonate transporter1
TIFY 4B: PEAPOD 2
AFPH2/NINJA: Novel interactor of JAZ
MYC2/JIN1: Jasmonate insensitive 1
AAO3: Abscisic-aldehyde oxidase
ZEP: Zeaxanthin epoxidase
ABA2: Xanthoxin dehydrogenase
ABA4: Neoxanthin synthase
NCED3: 9-cis-epoxycarotenoid dioxygenase
NRT1: Nitrate transporter 1
NRT2: Nitrate transporter 2
NRT3: Nitrate transporter 3
NRT4: Nitrate transporter 4
ABCG22: ATP-binding cassette G22
PYL8: PYR1-like8
PYL9: PYR1-like9
SRK2E: Serine/threonine-protein kinase
PP2C 16: Phosphatase 2C family 16
PP2C 24: Phosphatase 2C family 24
PP2C 37: Phosphatase 2C family 37
PP2C 75: Phosphatase 2C family 75
