# Supplementary material for: Comparative transcriptomic analyses of citrus cold-resistant vs. sensitive rootstocks might suggest a relevant role of ABA signaling in triggering cold scion adaption
Source: BMC Plant Biol. 2022 Apr 22;22:209. doi: 10.1186/s12870-022-03578-w (PMC9027863; doi:10.1186/s12870-022-03578-w)
Supplement: Supplementary file 1 — Additional file 1:Additional Figure 1. RNA-seq transcript validation of DEGs. Bars diagram indicates a relative TMMs from RNA-seq analysis and red line was the relative transcript gene level from RT-PCR. Figure a) DREB1D (LOC18033409), b) DREB1B (LOC18049462), c) PDH (LOC18039477), d) WCOR413 (LOC18035610), e) P5CS (LOC18044634), f) P5CDH (LOC18045924), g) NCED (LOC18046011) and h) PP2C (LOC18043434). Samples were measured in the leaves of the CAR and MAC plants grafted under cold (1°C) and control conditions for 0, 15 and 30 days. The values are the means±SE of three biological replicates (n=3) and three technical replicates per biological sample. The treatment effect tested by multi-way ANOVA, different letters indicate significant differences (P < 0.05) according LSD. [file 12870_2022_3578_MOESM1_ESM.pptx]

## Slide 1
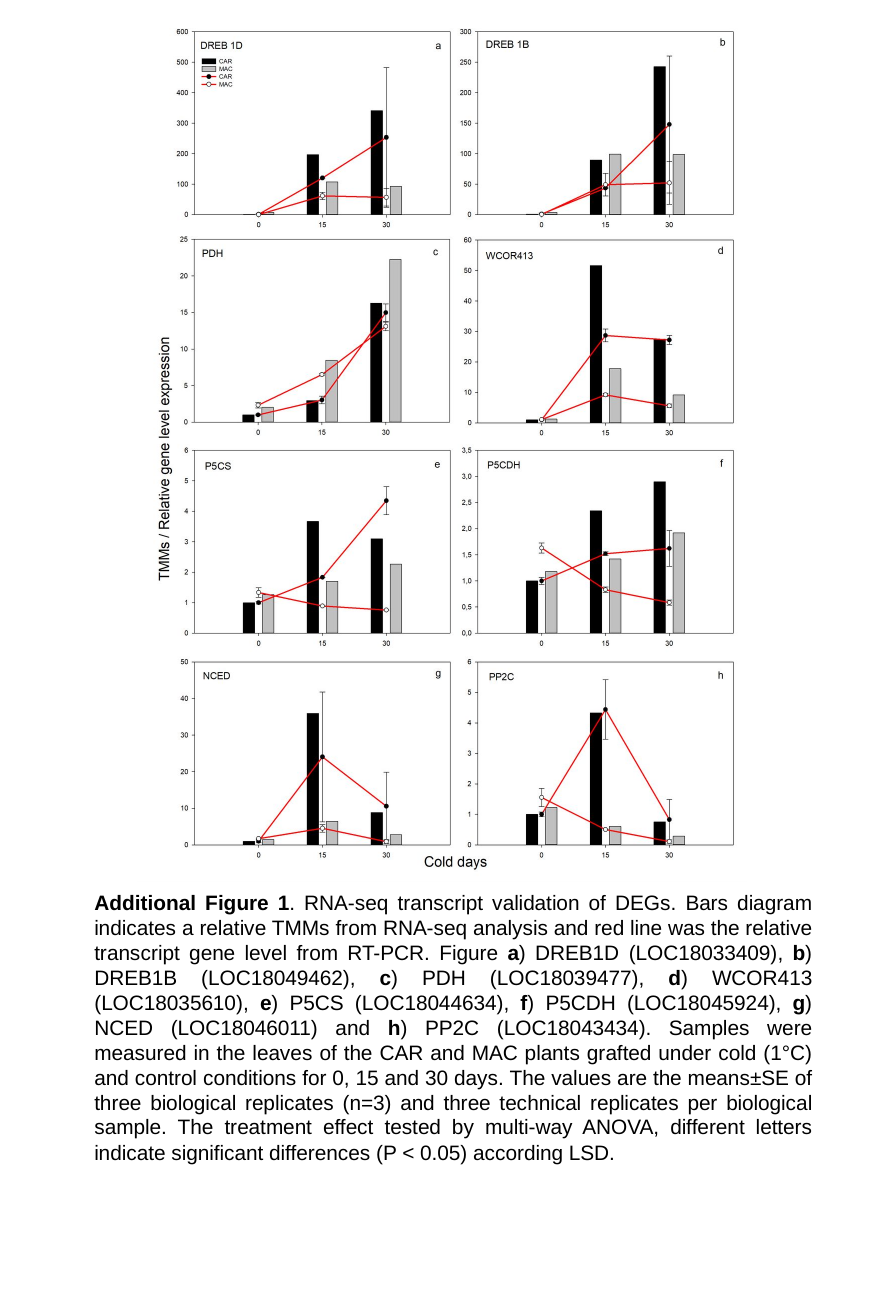

Additional Figure 1. RNA-seq transcript validation of DEGs. Bars diagram indicates a relative TMMs from RNA-seq analysis and red line was the relative transcript gene level from RT-PCR. Figure a) DREB1D (LOC18033409), b) DREB1B (LOC18049462), c) PDH (LOC18039477), d) WCOR413 (LOC18035610), e) P5CS (LOC18044634), f) P5CDH (LOC18045924), g) NCED (LOC18046011) and h) PP2C (LOC18043434). Samples were measured in the leaves of the CAR and MAC plants grafted under cold (1°C) and control conditions for 0, 15 and 30 days. The values are the means±SE of three biological replicates (n=3) and three technical replicates per biological sample. The treatment effect tested by multi-way ANOVA, different letters indicate significant differences (P < 0.05) according LSD.
