# Supplementary material for: Comparative transcriptomic analyses of citrus cold-resistant vs. sensitive rootstocks might suggest a relevant role of ABA signaling in triggering cold scion adaption
Source: BMC Plant Biol. 2022 Apr 22;22:209. doi: 10.1186/s12870-022-03578-w (PMC9027863; doi:10.1186/s12870-022-03578-w)
Supplement: Supplementary file 3 — Additional file 3:Additional Figure 2. Explanatory diagram of experimental procedure. [file 12870_2022_3578_MOESM3_ESM.pptx]

## Slide 1
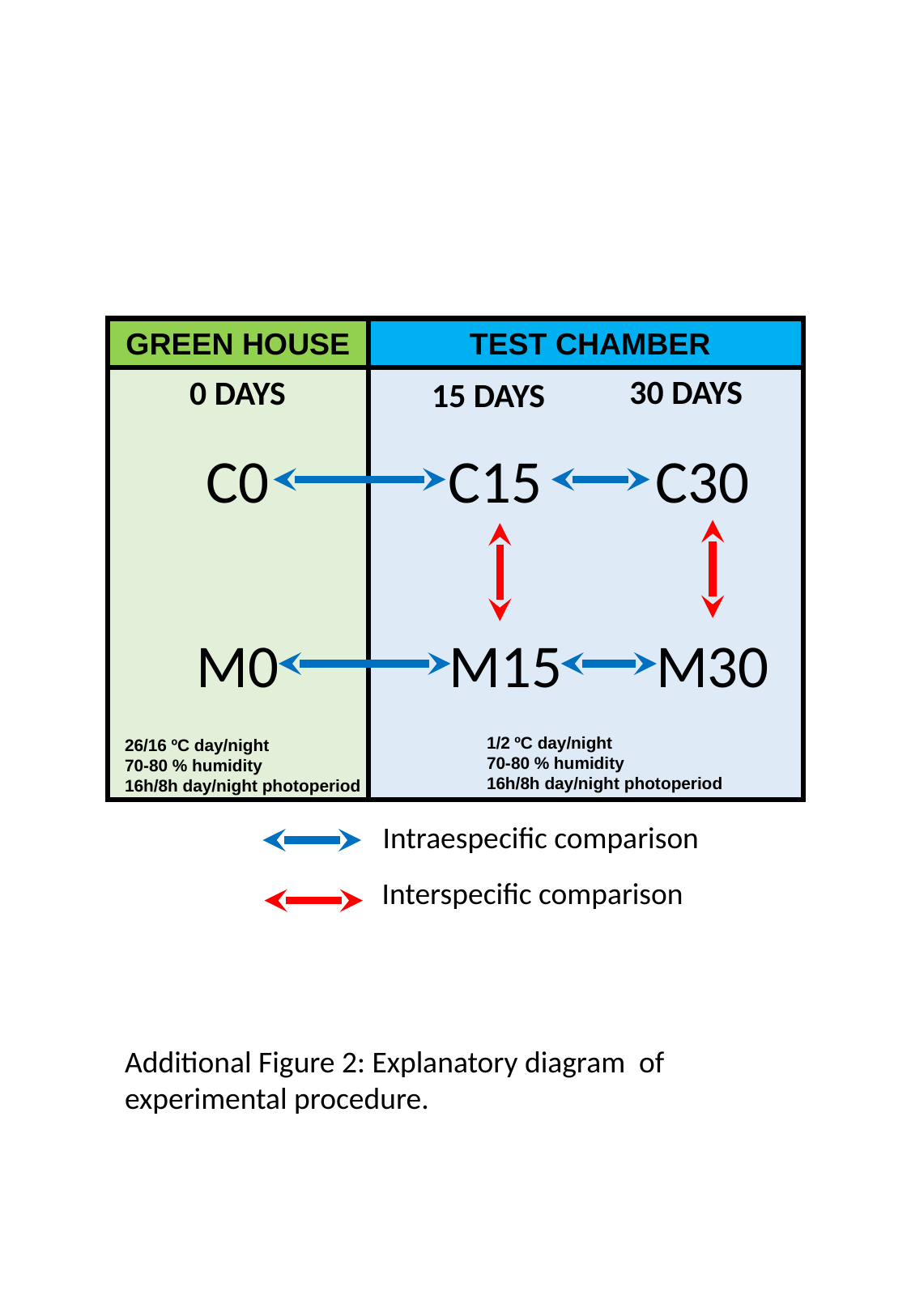

GREEN HOUSE
 TEST CHAMBER
30 DAYS
0 DAYS
15 DAYS
C0
C15
C30
M0
M15
M30
1/2 ºC day/night
70-80 % humidity
16h/8h day/night photoperiod
26/16 ºC day/night
70-80 % humidity
16h/8h day/night photoperiod
Intraespecific comparison
Interspecific comparison
Additional Figure 2: Explanatory diagram of experimental procedure.
