# Supplementary material for: Comparative transcriptomic analyses of citrus cold-resistant vs. sensitive rootstocks might suggest a relevant role of ABA signaling in triggering cold scion adaption
Source: BMC Plant Biol. 2022 Apr 22;22:209. doi: 10.1186/s12870-022-03578-w (PMC9027863; doi:10.1186/s12870-022-03578-w)
Supplement: Supplementary file 4 — Additional file 4:Additional Table 2. Raw data lectures in European Nucleotide Archive. [file 12870_2022_3578_MOESM4_ESM.doc]

Additional Table 2. Raw data lectures in European Nucleotide Archive.

| Std_accession | Sample_accession | Exp.accession | run_accession | tax_id | Scientific_name |
| --- | --- | --- | --- | --- | --- |
| PRJEB20758 | SAMEA104103360 | ERX2054017 | ERR1994148 | 105581 | Citrus sinensis x Citrus trifoliata |
| PRJEB20758 | SAMEA104055727 | ERX2023420 | ERR1960516 | 105581 | Citrus sinensis x Citrus trifoliata |
| PRJEB20758 | SAMEA104055728 | ERX2023421 | ERR1960517 | 307630 | Citrus macrophylla |
| PRJEB20758 | SAMEA104055729 | ERX2023422 | ERR1960518 | 307630 | Citrus macrophylla |
| PRJEB20758 | SAMEA104103361 | ERX2054018 | ERR1994149 | 105581 | Citrus sinensis x Citrus trifoliata |
| PRJEB20758 | SAMEA104103362 | ERX2054019 | ERR1994150 | 105581 | Citrus sinensis x Citrus trifoliata |
| PRJEB20758 | SAMEA104103363 | ERX2054020 | ERR1994151 | 307630 | Citrus macrophylla |
| PRJEB20758 | SAMEA104103364 | ERX2054021 | ERR1994152 | 307630 | Citrus macrophylla |
| PRJEB20758 | SAMEA104055094 | ERX2022792 | ERR1959283 | 105581 | Citrus sinensis x Citrus trifoliata |
| PRJEB20758 | SAMEA104103365 | ERX2054022 | ERR1994153 | 105581 | Citrus sinensis x Citrus trifoliata |
| PRJEB20758 | SAMEA104103366 | ERX2054023 | ERR1994154 | 307630 | Citrus macrophylla |
| PRJEB20758 | SAMEA104055736 | ERX2023423 | ERR1960519 | 307630 | Citrus macrophylla |
